# Student well-being: the impact of belonging, COVID-19 pandemic related student stress, loneliness, and academic anxiety

**DOI:** 10.3389/fpsyg.2025.1481328

**Published:** 2025-04-28

**Authors:** Gulsah Dost

**Affiliations:** School of Education, Durham University, Durham, United Kingdom

**Keywords:** COVID-19 pandemic, belonging, higher education, self-efficacy, loneliness, gender, anxiety, well-being

## Abstract

**Introduction:**

This research investigates the relationship between collegiate community and degree department belonging, loneliness, COVID-19 pandemic related student stress, coping self-efficacy, and academic anxiety among UK collegiate context. The study focuses on how these factors have shaped students’ academic and social experiences in the post-pandemic era, with particular emphasis on variations based on gender as well as home versus international status among both undergraduate and postgraduate cohorts.

**Methods:**

This study utilized structural equation modelling (SEM). A sample of 430 students was administered, with 284 (66%) completed by female students and 120 (28%) by male students. The number of undergraduate students was (*N* = 244, 56.7%), and (*N* = 184, 42.8%) participants were postgraduate students.

**Results:**

The research findings indicate that stress related to COVID-19 has adversely affected both types of belongingness— collegiate community and degree department belonging —while concurrently elevating academic anxiety across diverse demographic groups. Loneliness emerged as a significant mediating variable, with pronounced effects observed among international and male students. The presence of academic anxiety exacerbated feelings of loneliness and diminished coping self-efficacy, particularly in female and undergraduate cohorts. International students experienced notable disruptions in their sense of collegiate belonging and heightened levels of loneliness. Undergraduate students demonstrated greater susceptibility to stress-induced declines in belongingness, whereas postgraduate students reported more severe ramifications of loneliness on their academic and social relationships.

**Discussion:**

These results underscore the necessity for targeted interventions designed to foster social connectedness, alleviate academic anxiety, and bolster support systems within the post-pandemic educational framework.

## 1 Introduction

In the field of education, the concept of “belonging” is highly significant, as it relates closely to how students perceive their connection to their educational institutions. This notion of belonging encompasses the degree to which students feel they are accepted, respected, included, and supported within their academic environments (Dost, 2024a). The framework of belonging emphasises the essential role of interpersonal relationships and social integration in enhancing the educational journey. These elements not only improve educational experiences but also drive student engagement and foster academic success (Goodenow, 1993). Belonging is recognised as a fundamental human motivation crucial for overall well-being. It embodies the need for frequent social interactions and entails the perception of stable and emotionally significant interpersonal connections that endure over time (Baumeister and Leary, 1995). Within the context of higher education, belonging captures the essence of how connected students feel to both their campus community and their specific academic departments. Dost and Mazzoli Smith (2023) provide an insightful definition of university belonging. They describe it as “feeling part of somewhere an individual can be themselves and feel confident in their personal and social identities, through secure, meaningful, and harmonious support in cohesion with other diverse group members and creating ethnically heterogeneous communities and learning areas both on and off the faculty/campus settings” (p. 841). This characterisation highlights the multifaceted nature of belonging, which involves not only personal authenticity but also the establishment of supportive relationships across diverse populations. Sense of belonging occurs when a person feels valued, respected, and welcomed by other members of the group/community of which they are a part (Dost, 2024a; Dost, 2024b). Especially when evaluated within the context of higher education, a sense of belonging allows students to connect with their institutions, to feel a part of society, and to feel respected for their abilities and characteristics (Strayhorn, 2018). Feeling welcomed, valued, and respected not only by the community/group to which they belong, but also by other members in cases where there are common bridges between groups, has a positive effect on students’ pride in their work, their active participation, productivity and, eventually their belonging (Dost, 2024a). When students genuinely feel connected and included in their college environment and academic departments, they become more engaged in both academic and social activities (Pedler et al., 2022). This increased engagement often leads students to actively seek support from their peers, department and college communities, as well as to utilise various resources available on department or college, such as tutoring services, counselling, and extracurricular activities (Nunn, 2021; Strayhorn, 2018).

The concept of collegiate community belonging represents a pivotal aspect of the collegiate experience, embodying a profound sense of connection, acceptance, and inclusion that significantly influences students’ trajectories through higher education. Collegiate universities in the United Kingdom (UK) differ from non-Collegiate universities in that they are composed of semi-autonomous colleges, each of which operates its own dining services, residential accommodations, communal facilities, library resources, athletic amenities, scholarship programs, and student support services (University of Cambridge, 2024; University of Oxford, 2024a). Furthermore, they feature structured social environments through junior common rooms (JCR), middle common rooms (MCR), and senior common rooms (SCR), all overseen by a head, typically designated as the master or principal of the college (University of Durham, 2024). Within the framework of a collegiate system, students have the opportunity to cultivate a close-knit and supportive community within their respective colleges. Rather than being a mere adjunct to the college experience, collegiate community belonging serves as a foundational element, contingent upon critical factors such as acceptance, respect, and continuous support from diverse constituents within the college community, including peers, department, and administrative staff (University of Oxford, 2024b). These elements are crucial in enhancing the student experience and fostering an environment where individuals feel valued and integrated into the institution’s academic and social dynamics. While collegiate community belonging captures a comprehensive sense of community and inclusivity, it plays a vital role in alleviating the loneliness often encountered by students. A robust sense of college belonging can function as a buffer against feelings of isolation, concurrently enhancing mental health and overall well-being (University of Oxford, 2024b). On the other hand, degree department belonging specifically addresses students’ perceptions of their roles and statuses within their academic departments (Strayhorn, 2018; Zahl, 2015). Department belonging underscores the significance of feeling accepted and supported within one’s discipline, which is essential for promoting engagement and participation in discipline-specific activities (Dost and Mazzoli Smith, 2023; Wilson et al., 2015). A strong sense of department belonging offers immediate support that can mitigate academic anxiety, encouraging student involvement in scholarly endeavours and department-specific initiatives (Ahn and Davis, 2020; Pedler et al., 2022; Romeo et al., 2024).

A significant correlation exists between heightened levels of college and department belonging and diminished levels of academic anxiety and loneliness (Chen et al., 2022; Pittman and Richmond, 2008; Singh et al., 2021; Vasileiou et al., 2019; Zhu et al., 2019). Academic anxiety is a prevalent concern among students, often leading to symptoms of loneliness (Tan et al., 2023). This arises because students experiencing intense anxiety may retreat from social interactions as a coping mechanism to handle the pressures of academic demands, which may, in turn, exacerbate feelings of isolation and dilute opportunities for forming supportive relationships critical for emotional and psychological resilience (Freyhofer et al., 2021; Singh et al., 2021; Zhang et al., 2024). Moreover, the relationship is bidirectional: loneliness can amplify academic anxiety by undermining emotional resilience and increasing vulnerability within academic contexts (Phillips et al., 2022; Renati et al., 2023). Students exhibiting high coping self-efficacy—defined as the belief in one’s capability to manage stress effectively—tend to navigate academic challenges more adeptly, resulting in mitigated academic anxiety and its associated negative impacts on well-being (Schunk and DiBenedetto, 2020, 2022). This heightened confidence not only alleviates feelings of loneliness but also fosters proactive social engagement and problem-solving strategies. Moreover, individuals with increased coping self-efficacy exhibited lower levels of anxiety and depression during the pandemic (Arora et al., 2021). The enhancement of coping self-efficacy has proven instrumental in alleviating academic stressors, particularly in the context of the transition to online learning and the prevailing uncertainties regarding future career trajectories (Wong and Yuen, 2023). In periods of social isolation, individuals with higher coping self-efficacy demonstrated a greater ability to maintain emotional equilibrium and engage in proactive coping strategies, such as virtual social interactions and the pursuit of recreational activities (Cattelino et al., 2023; Freire et al., 2020).

The impact of the COVID-19 pandemic on students in the UK has exhibited significant complexity, and findings related to the pandemic and post-pandemic periods reveal a range of mixed results regarding students’ experiences and outcomes. For example, Chen and Lucock (2022) indicated that over 50% of 1,173 university students from Northern England displayed anxiety and depression levels exceeding clinical thresholds. Similarly, Liverpool et al. (2023) investigated the mental health and well-being of students in further and higher education following the resumption of face-to-face learning after COVID-19 restrictions. However, the results revealed moderate levels of anxiety and depression, with well-being scores significantly lower than pre-pandemic benchmarks. Additionally, Meda et al. (2021) reported a marked increase in depression levels among participants during the pandemic, while anxiety levels appeared to remain stable. Conversely, Saraswathi et al. (2020) noted heightened anxiety levels among undergraduate students without a similar rise in depressive symptoms during the same timeframe. This multifaceted trend has been reflected in the sense of belonging, feelings of loneliness and coping self-efficacy among students. Research by Ouzia et al. (2023) revealed that students who began their studies during the height of the pandemic or shortly after experienced notably lower levels of belonging compared to those who started before the pandemic. However, a survey carried out by Blake et al. (2022) found a measure of resilience, with 69% of respondents from 15 UK universities reporting a sense of belonging by late 2021. Furthermore, Schochet et al. (2023) emphasised that feelings of academic distress and loneliness had risen significantly during the pandemic compared to pre-pandemic times. In addition, Weber et al. (2022) noted a marked increase in loneliness reports throughout the pandemic, closely associated with symptoms of anxiety and depression.

Despite an increase in reported stress, some studies indicate that stress levels remained similar to those before the pandemic. King (2022) found that while postgraduate research students at a Welsh university reported low stress, they experienced moderate loneliness during the pandemic. Research highlights that students with lower coping self-efficacy (CSE) struggle more with feelings of isolation and stress management (Mahoney and Benight, 2019). Fluharty et al. (2020) observed a decrease in mental distress over 21 weeks as lockdown measures eased, although most coping strategies did not correlate significantly with this decline. The existing literature on student mental health during the post-pandemic phase is limited, highlighting the need for further research to inform effective support systems. This study aims to create a comprehensive framework that integrates stress-appraisal theory (Lazarus and Folkman, 1984), belongingness theory (Baumeister and Leary, 1995), and self-efficacy theory (Bandura, 1997) to better understand the complex impacts of COVID-19-related stress on student well-being. Unlike previous research that tends to focus on isolated aspects of the student experience, this investigation presents a holistic model that connects various factors such as stress, feelings of belonging, loneliness, coping self-efficacy, and academic anxiety. Rather than simply listing unconnected variables, the study integrates them into a unified theoretical framework that clarifies the reasons and mechanisms behind these relationships. By examining these interrelated dimensions, the research reveals how COVID-19 exacerbated existing challenges and created new ones, affecting students’ emotional and academic lives. The choice of key variables for this study is supported by well-established psychological and educational theories that relate to student well-being, persistence in academics, and mental health. The justification for selecting these particular variables lies in their importance to how students adjust during their time in higher education and their significance in light of the disruptions caused by the pandemic. By concentrating on these theoretically and empirically supported factors, this study offers a unified, evidence-based framework for comprehending student adjustment in the post-pandemic period. The focus is on undergraduate and postgraduate students at a UK university that employs a collegiate system. This structure is particularly significant as it fosters a strong sense of belonging among students, not only to their respective colleges but also to the broader university community (Strayhorn, 2018). Students who identify as members of their department, college, and academic or athletic teams frequently report notable reductions in feelings of loneliness and academic stress (Haslam et al., 2009). Furthermore, this sense of connectedness plays a vital role in enhancing their coping self-efficacy skills, which are essential for effectively navigating the challenges associated with university life. By exploring these dynamics, the study seeks to provide valuable insights into how the cultivation of a sense of community within colleges and degree departments can positively influence students’ experiences throughout their university journey. The findings are expected to provide valuable insights for higher education policymakers, highlighting crucial intervention points that can be targeted to alleviate the long-term effects of the pandemic.

### 1.1 Research hypotheses

#### 1.1.1 The impact of the COVID-19 pandemic-related stress on collegiate community belonging, department belonging, loneliness, academic anxiety, and coping self-efficacy in the post-pandemic period

The COVID-19 pandemic introduced a series of unprecedented stressors that significantly affected the academic and social experiences of students across all levels of higher education (Burns et al., 2020). As institutions rapidly transitioned from in-person instruction to online learning environments, students encountered considerable challenges. This abrupt disruption critically compromised students’ sense of belonging and their coping mechanisms, resulting in a notable increase in their pandemic related stress and decline in mental health and overall well-being (Pasupathi et al., 2022). The stress-appraisal model (Lazarus and Folkman, 1984) defines stress as emerging from a person’s view that the demands of their environment surpass their available resources. The stressors associated with COVID-19, such as interruptions in education, feelings of social isolation, financial instability, and health worries, significantly impacted students’ ability to cope, resulting in negative effects on their education and mental well-being (Son et al., 2020). The stress induced by the pandemic has the potential to weaken individuals’ sense of belonging not only within their specific academic departments but also within the broader institutional framework (Barringer et al., 2023). The shift away from traditional face-to-face interactions, which are vital for relationship-building and community fostering, engendered a sense of distance and isolation among students and departments alike (Heider, 2021). This alteration, combined with disruptions to established academic practices such as collaborative learning, mentorship opportunities, and informal social gatherings, hindered the formation of robust academic and social networks (Leal Filho et al., 2021). Consequently, many students found it increasingly difficult to navigate their educational pathways effectively, as they lost crucial resources and relationships that previously facilitated their academic journeys (Bozkurt et al., 2020; Sato et al., 2023). As a result, numerous individuals experienced heightened challenges in connecting with peers and colleagues, which can exacerbate feelings of loneliness and disengagement within an already stressful environment (Hamza et al., 2021).

During the pandemic, a considerable number of students reported pervasive feelings of isolation, loneliness, and disconnection from their academic communities, which led to diminished engagement and a weakened sense of belonging within their respective departments, colleges, or institutions (Bierman et al., 2021; Dost, 2024b). In the post-pandemic landscape, Kathirvel (2020) underscored the likelihood that the mental health consequences arising from the COVID-19 pandemic will extend well beyond the immediate crisis, potentially affecting individuals over several years. Jamshaid et al. (2023) also evaluated the mental health of international students prior to the onset of the pandemic, revealing that this demographic generally reported positive mental health. However, as the pandemic evolved, there was a notable increase in symptoms of depression and anxiety within this population. These findings underscore a concerning trend that presents significant risks to the well-being of students during the post-pandemic period, particularly as they navigate the complexities of acclimatising to a new cultural environment. Compounding these challenges, pandemic-related stressors such as health-related and academic anxieties, financial insecurities, and worries regarding future employment prospects have notably weakened students’ coping mechanisms (Haikalis et al., 2022; Hamaideh et al., 2022; Wang and Cheng, 2020). Self-Efficacy Theory (Bandura, 1997) indicates that individuals who possess high levels of coping self-efficacy are more capable of managing stress effectively. Students with higher self-efficacy showed greater resilience in adapting to pandemic-related challenges, displaying enhanced persistence, problem-solving abilities, and emotional regulation (Hamaideh et al., 2022). In contrast, individuals with reduced self-efficacy experienced increased levels of stress, anxiety, and depression, which subsequently hindered their academic performance and overall quality of life (Hamza et al., 2021). These mental health challenges not only affect students’ academic performance but also have lasting implications for their overall quality of life. The following hypotheses are proposed to highlight the impact of the COVID-19 pandemic-related stress during the post-pandemic period on collegiate community belonging, department belonging, loneliness, academic anxiety, and coping self-efficacy:

*Hypothesis 1:* COVID-19 pandemic-related stress will have a negative impact on degree department belonging among undergraduate and postgraduate students.

*Hypothesis 2:* COVID-19 pandemic-related stress will have a negative impact on coping self-efficacy among undergraduate and postgraduate students.

*Hypothesis 3:* COVID-19 pandemic-related stress will have a negative impact on loneliness among undergraduate and postgraduate students.

*Hypothesis 4:* COVID-19 pandemic-related stress will have a negative impact on collegiate community belonging among undergraduate and postgraduate students.

*Hypothesis 5:* COVID-19 pandemic-related stress will have a negative impact on academic anxiety among undergraduate and postgraduate students.

#### 1.1.2 The impact of loneliness on degree department belonging, collegiate community belonging, and coping self-efficacy

Loneliness is a significant psychological factor and is characterised by feelings of social isolation and a lack of meaningful relationships, which can profoundly impact students’ sense of belonging within their academic departments and college communities, affecting both undergraduate and postgraduate students (Allen, 2020; Heinrich and Gullone, 2006; Matook et al., 2015). Baumeister and Leary's (1995) belongingness theory posits that humans have an intrinsic need to form and maintain strong interpersonal bonds. When individuals are unable to cultivate these connections, they may experience emotional turmoil that could manifest as distress and increased feelings of alienation (Cacioppo and Patrick, 2008). This emotional state can, in turn, lead to a marked decrease in active participation in social or academic spheres, creating a cycle of isolation (Walsham et al., 2023). The Cognitive Discrepancy Model of Loneliness (Peplau and Perlman, 1982) argues that loneliness arises when an individual’s social expectations are unmet. Students who reported heightened levels of loneliness often exhibited lower motivation to engage in departmental activities, which can include everything from attending college activities or lectures to participating in study groups and departmental or college events (Diehl et al., 2018; Thomas, 2012). The issue of loneliness can be particularly pronounced for postgraduate students, who frequently encounter unique challenges, such as working in isolated research settings (Janta et al., 2014; King, 2022). Unlike undergraduates, who often have more structured opportunities for social interaction through class activities, postgraduate students may find themselves in environments where colleague interaction is limited. Loneliness also negatively impacts coping self-efficacy by fostering feelings of isolation and insecurity, which ultimately undermines students’ confidence in their ability to navigate both academic and personal challenges (Icekson et al., 2021; Vasileiou et al., 2019). This decline in self-assurance regarding coping mechanisms may further distance students from their peer networks and academic relationships. By promoting resilience and adaptability, coping self-efficacy provides students with essential skills and strategies to effectively navigate difficult emotional states (Icekson et al., 2021). This capability not only assists them in confronting immediate challenges but also fosters a sense of belonging within their academic departments and college communities (Raymond and Sheppard, 2018; Trujillo and Tanner, 2014). Furthermore, it prepares them for future adversities, enabling them to approach life’s uncertainties with confidence and strength (Cattelino et al., 2023; Navickienė and Vasiliauskas, 2024). As a result, loneliness is likely to represent a significant obstacle in promoting coping self-efficacy, as well as a sense of community and identity within academic departments and college environments (Allen et al., 2021; Chipchase et al., 2017). This absence of connection can contribute to increased disengagement from academic activities and adversely affect students’ overall sense of belonging within their respective departments and college experiences. Therefore, the following hypotheses are proposed to address the impact of loneliness on department and collegiate community belonging:

*Hypothesis 6:* Loneliness will have a negative impact degree department belonging among undergraduate and postgraduate students.

*Hypothesis 7:* Loneliness will have a negative impact on collegiate community belonging among undergraduate and postgraduate students.

*Hypothesis 8:* Loneliness will have a negative impact on coping self-efficacy among undergraduate and postgraduate students.

#### 1.1.3 The effect of collegiate community belonging on coping self-efficacy

The concept of belonging within a collegiate environment is likely to be integral to the development of students’ coping self-efficacy, which encompasses their belief in their capability to effectively manage both academic challenges and personal difficulties (Freire et al., 2020). According to belongingness theory (Baumeister and Leary, 1995), a profound sense of belonging cultivates strong social connections, emotional support, and access to diverse resources, all of which collectively enhance students’ perceptions of their ability to navigate and cope with the stressors encountered throughout their college experience. Students who foster a robust sense of connection to their college communities are more likely to view their environment as supportive and inclusive. This favourable perception significantly influences their confidence in handling academic responsibilities and social relationships (Walton and Cohen, 2011). When students experience a sense of belonging, they encounter heightened emotional security and a more defined sense of identity, which enables them to devote increased focus to building resilience and developing effective coping strategies (Au et al., 2023; Cahill et al., 2014; Nowicki, 2008). Moreover, when students feel accepted and valued within their college environments, they are more prone to engage actively with their peers, seek assistance when necessary, and utilise institutional resources designed to facilitate their success. This proactive engagement leads to improvements in their coping self-efficacy (Strayhorn, 2018). Conversely, a lack of belonging can substantially undermine students’ confidence, foster feelings of isolation, and detract from their self-efficacy (Strayhorn, 2018). Such adverse experiences may negatively affect their academic performance and overall emotional well-being. The intricate relationship between a sense of belonging and coping self-efficacy underscores the imperative for educational institutions to cultivate inclusive and supportive campus atmospheres (Jordan, 2014). In light of this understanding, the following hypothesis is proposed to rigorously investigate the influence of collegiate community belonging on self-efficacy:

*Hypothesis 9:* Collegiate community belonging will have a positive effect on coping self-efficacy among undergraduate and postgraduate students.

#### 1.1.4 The effect of coping self-efficacy on department belonging

Coping self-efficacy, conceptualised as the belief in one’s capability to navigate and surmount stressors effectively, is fundamental in shaping the experiences of undergraduate and postgraduate students within academic settings (Chesney et al., 2006; Freire et al., 2020). High coping self-efficacy correlates with reduced feelings of loneliness, enabling students to engage proactively in social interactions (Icekson et al., 2021). Heinrich and Gullone (2006) indicate that individuals who perceive themselves as capable of effective coping are more likely to seek social support, initiate interpersonal connections, and tackle social challenges more competently. This enhanced social engagement mitigates feelings of isolation and promotes the establishment of significant relationships, thereby augmenting students’ overall sense of belonging within their academic departments (Trujillo and Tanner, 2014). Students with elevated self-efficacy often display enhanced confidence in their interactions with peers and faculty, facilitating participation in collaborative endeavours, mentorship relationships, and departmental activities (Basson, 2021; Craig, 2018; Lamssali et al., 2024; Reid, 2013). Developing coping self-efficacy equips students to adeptly manage academic demands, forge meaningful connections, and nurture a strong sense of belonging within their educational contexts (Dantzler, 2023; Freire et al., 2020). Consequently, the following hypothesis is proposed to address the effect of coping self-efficacy on department belonging:

*Hypothesis 10:* Coping self-efficacy will have a positive effect on degree department belonging among undergraduate and postgraduate students.

#### 1.1.5 The impact of academic anxiety on loneliness, coping self-efficacy, degree department and collegiate community belonging

Academic anxiety significantly contributes to feelings of loneliness across various stages of higher education, encompassing undergraduate and postgraduate students (Hamdan-Mansour et al., 2024). The psychological construct of academic anxiety significantly influences key dimensions of student life, notably coping self-efficacy, loneliness, and the sense of belonging to one’s college and degree department community. A positive sense of belonging within an academic department, which is characterised by feelings of connection and support, can unintentionally lead to increased academic anxiety among students. This phenomenon occurs as students internalise the expectations and pressures for success imposed by their department (Ramos-Sánchez and Nichols, 2007). For example, students who feel a deep connection to their department may feel compelled to meet high standards of performance, leading to stress and anxiety about academic outcomes (Chemers et al., 2001; Hurtado and Carter, 1997; Richardson et al., 2012). When students experience heightened anxiety related to their academic performance, deadlines, or expectations, it can diminish their confidence in their ability to effectively manage stress and succeed in their studies. This decline in self-efficacy often creates a cyclical pattern in which students’ anxiety leads to decreased engagement with academic pursuits, thereby exacerbating feelings of inadequacy (Bandura, 1997). In addition, the nexus between heightened academic anxiety and social isolation is well-documented, arising from the intense fear of failure, excessive performance pressure, and overwhelming workloads that characterise students’ experiences (Archbell and Coplan, 2022; Ajjawi et al., 2020; Bergin and Pakenham, 2015). These elements often lead to social withdrawal, as individuals prioritise academic responsibilities over interpersonal engagement or grapple with embarrassment stemming from their struggles (Beiter et al., 2015). This withdrawal creates a detrimental feedback loop, exacerbating feelings of loneliness and further discouraging social participation.

For undergraduates, the transition into a new academic environment introduces unique stressors, including the challenge of adapting to unfamiliar expectations (Leary and DeRosier, 2012). This adaptation phase can significantly impede their capacity to form connections and cultivate lasting relationships (Conley et al., 2014; Dost, 2024a), particularly during the early stages of their collegiate experience. In contrast, postgraduate students, particularly those in doctoral programs, encounter a form of academic anxiety that is isolating yet distinct. The pressure to achieve research milestones, adhere to looming deadlines, and contemplate future career trajectories can be overwhelming. Unlike undergraduate students, doctoral candidates typically navigate a less structured academic landscape, which often lacks robust social frameworks facilitating interaction and camaraderie (Erichsen and Bolliger, 2011; Sverdlik et al., 2018). This absence can aggravate feelings of isolation among doctoral students. Conversely, college belonging may alleviate academic anxiety by fostering a close-knit network of peers, mentors, and faculty who provide emotional and academic support (Strayhorn, 2018). This sense of belonging helps students navigate academic challenges more effectively, reducing feelings of isolation and uncertainty (Antonsich, 2010). Particularly for postgraduate students, who often experience solitary research environments, department belonging can provide essential scaffolding to mitigate anxiety related to academic tasks and career pressures (Evans et al., 2018). Therefore, while department belonging can increase academic anxiety due to heightened pressures, college belonging serves as a critical buffer that helps students manage academic demands. Encouraging students to perceive their anxiety as a manageable challenge while fostering a supportive academic and collegiate environment can effectively interrupt the cycle of anxiety and assist students in rebuilding their confidence in their ability to cope. Addressing these interconnected issues is vital for cultivating a more inclusive and supportive academic environment, ultimately mitigating academic anxiety and the associated loneliness experienced by students. Consequently, the following hypotheses are proposed:

*Hypothesis 11:* Academic anxiety will have a negative impact on collegiate community belonging among undergraduate and postgraduate students.

*Hypothesis 12:* Academic anxiety will have a negative impact on degree department belonging among undergraduate and postgraduate students.

*Hypothesis 13:* Academic anxiety will have a negative impact on coping self-efficacy among undergraduate and postgraduate students.

*Hypothesis 14:* Academic anxiety will have a positive impact on loneliness among undergraduate and postgraduate students.

#### 1.1.6 The relationships among the variables and diverse demographic and academic groups

The interplay among collegiate community belonging, COVID-19-related student stress, department belonging, loneliness, coping self-efficacy, and academic anxiety exhibits notable complexity and variability across diverse demographic and academic cohorts (i.e., gender identity, educational level, and whether the students are international or home status). Existing research underscores the variable intersections of these factors, revealing distinct challenges endemic to each group. For instance, gender has been identified as a pivotal determinant in shaping stress responses and coping strategies (Graves et al., 2021). Female and non-binary students report elevated levels of academic anxiety and loneliness (McKinney, 2021; McNamara, 2021). Male students also displayed an emotional landscape characterised by a stronger connection between stress and loneliness, with their reliance on limited and less diverse support networks leading to increased isolation (Essadek and Rabeyron, 2020). Undergraduates, situated in critical phases of personal and academic development, often depend on their sense of belonging within the collegiate ecosystem to alleviate stress (Bentrim and Henning, 2023; Strayhorn, 2018). Their pronounced need for social connections may render them particularly susceptible to loneliness when confronted with academic challenges. In contrast, doctoral students face intensified pressures associated with research demands and career trajectory uncertainties, which can exacerbate academic anxiety (Acharya et al., 2024). This demographic frequently navigates high-stakes expectations surrounding original research output, significantly impacting their overall mental health (Lovitts, 2001). International students contend with a distinctive array of stressors that amplify their challenges. They navigate considerable cultural transitions, language barriers, and isolation from known socio-cultural contexts. These factors can intensify feelings of loneliness and erode their departmental belonging, especially in comparison to their domestic counterparts (Smith and Khawaja, 2011). The effectiveness of support systems accessible to international students plays a critical role in shaping their coping strategies and self-efficacy, particularly given that they may encounter limited resources or networks relative to home students (Lian et al., 2020; Sabouripour and Roslan, 2015). In conclusion, the intricate dynamics linking college belonging, pandemic stress, and academic anxiety highlight the imperative for targeted interventions customised to distinct demographic and academic cohorts. Therefore, the following hypotheses are assumed:

*Hypothesis 15:* The associations among collegiate community belonging, COVID-19-related student stress, degree department belonging, loneliness, coping self-efficacy, and academic anxiety will differ among female and male students.

*Hypothesis 16:* The associations among collegiate community belonging, COVID-19-related student stress, degree department belonging, loneliness, coping self-efficacy, and academic anxiety will differ across undergraduate and postgraduate students.

*Hypothesis 17:* The associations among collegiate community belonging, COVID-19-related student stress, degree department belonging, loneliness, coping self-efficacy, and academic anxiety will differ across international and home students.

**Figure 1:**
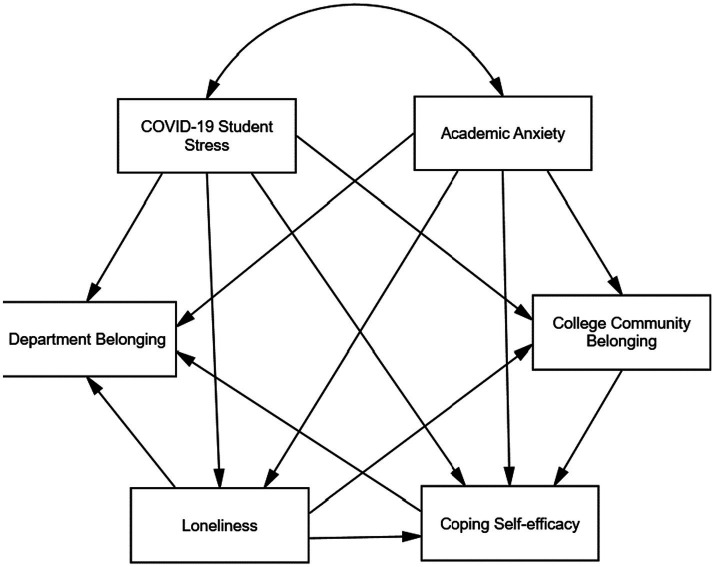
Theoretical model.

The research hypotheses are diagrammed in the theoretical model presented in Figure 1.

## 2 Methodology

### 2.1 Purpose of the study and research hypotheses

This study employed confirmatory factor analysis (CFA), structural equation modelling (SEM), and multi-group structural equation modelling (MGSEM) to first investigate the relationships between collegiate community belonging, COVID-19 pandemic student stress, degree department belonging, loneliness, coping self-efficacy, and academic anxiety. Secondly, this research aimed to investigate the impact of socio-demographic factors, including gender, education level, and international and home student status, on the correlations between the variables among undergraduate and postgraduate students at a UK university with a collegiate system. A conceptual model based on existing literature was developed to depict the interrelations among these factors (see Figure 1). To validate the model, the study addressed 17 research hypotheses.

### 2.2 Recruitment and participants

The data collection utilised the convenience sampling method. Due to the constraints of time and available resources, this study employed a convenience sampling method. This approach allowed for the efficient collection of data from participants who were easily accessible, facilitating a quicker conclusion to the research process (Lopez and Whitehead, 2013). While utilising a stratified sampling method might have resulted in a more accurately representative sample, practical limitations made this option impractical. To enhance the validity of the findings and mitigate potential biases associated with convenience sampling, several measures were taken. Notably, the sample included a diverse group of participants that reflected critical demographic variables such as age, gender, and socio-economic status. This careful selection aimed to mirror the characteristics of the larger population, thereby improving the generalisability of the results. Moreover, standardised tools and instruments for data collection were employed to ensure consistency and reliability across the gathered information. By focusing on these key aspects, the study aimed to uphold the integrity of the research despite the inherent limitations of the sampling method used.

Data was gathered over the course of the first and second terms of 2024, each spanning a one-month period, using questionnaires. Throughout this timeframe, students were sent three reminders. Gatekeepers facilitated the distribution of survey links through email to students at a Russell Group^1^ University that operates with a collegiate system. Recruitment of participants was done through flyers and email, with gatekeepers aiding in the distribution of emails to students. The research invitation emails were sent to undergraduate and postgraduate students at the Russell Group University with a collegiate system. Participation in the study was voluntary and anonymous, and students provided written consent. A sample size of 200 or larger is typically recommended for SEM analysis (Boomsma, 1982; Kyriazos, 2018). In the context of multi-group analysis in SEM, a commonly accepted guideline is to aim for a minimum of 100 cases or observations per group (Kline, 2005). This threshold helps ensure robust statistical power and reliable parameter estimates across different groups. A total of 430 questionnaires were disseminated in this study, with 284 (66%) completed by female students, 120 (28%) by male students, and 21 (5%) by non-binary students. However, non-binary respondents were excluded from the analysis due to not meeting the minimum threshold required for MGSEM. The majority of participants were in the 18–23 age range (*N* = 318, 74%), followed by the 24–29 age group (*N* = 83, 19.3%). The largest ethnic group among participants was White (*N* = 171, 39.8%), followed by Asian British-Chinese (*N* = 86, 20%), then by participants from any other White backgrounds (*N* = 64, 14.9%), and lastly by participants from other Asian backgrounds (*N* = 26, 6%). The number of undergraduate students (*N* = 244, 56.7%) was twice as high as the number of master’s students (*N* = 112, 26%), with 72 (16.8%) participants being doctorate students. The highest number of students were in their first year (*N* = 177, 41.2%), followed by second-year students (*N* = 107, 24.9%), with 100 (23.3%) students in their third year and 39 (9.1%) students in their fourth year. There were 219 (50.9%) students with home student status and 208 (48.4%) international students. The number of first-generation students was 120 (27.9%), and the number of non-first-generation students was 292 (67.9%). The demographic information of students can be found in Table 1.

**Table 1:** Demographic characteristics of respondents (*N* = 430).

Variables	Female (*N* = 284)	% (66%)	Male (*N* = 120)	% (28%)	Non-binary (*N* = 21)	% (5%)	Prefer not to say (*N* = 5)	% (1%)	*N*	%
Age										
18–23 years old	210	73.9%	89	74.2%	16	76.2%	3	60.0%	318	74.0%
24–29 years old	55	19.4%	23	19.2%	5	23.8%			83	19.3%
30–35 years old	9	3.2%	2	1.7%			2	40.0%	13	3.0%
36–41 years old	2	0.7%	1	0.8%					3	0.7%
42 years old and above	8	2.8%	5	4.2%					13	3.0%
Ethnicity										
White-English/British/ Welsh/ Scottish/ Northern Irish	114	40.1%	45	37.5%	11	52.4%	1	20.0%	171	39.8%
White-Irish	2	0.7%	2	1.7%					4	0.9%
Any other White background	36	12.7%	23	19.2%	4	19.0%	1	20.0%	64	14.9%
Mixed/multiple-White and Black Caribbean	1	0.4%	1	0.8%					2	0.5%
Mixed/multiple-White and Asian	9	3.2%	4	3.3%					13	3.0%
Any other Mixed or Multiple ethnic background	6	2.1%	1	0.8%			2	40.0%	9	2.1%
Asian/Asian British-Indian	12	4.2%	1	0.8%	1	4.8%			14	3.3%
Asian/Asian British-Pakistani	2	0.7%	5	4.2%					7	1.6%
Asian/Asian British-Bangladeshi	1	0.4%							1	0.2%
Asian/Asian British-Chinese	65	22.9%	18	15.0%	3	14.3%			86	20.0%
Any other Asian background	18	6.3%	6	5.0%	2	9.5%			26	6.0%
Black and Black British-African	1	0.4%	3	2.5%					4	0.9%
Prefer not to say	10	3.5%	6	5.0%			1	20.0%	17	4.0%
Other	7	2.5%	5	4.2%					12	2.8%
Education level										
Bachelor’s degree (e.g., BA, BSc)	165	58.1%	64	53.3%	14	66.7%	1	20.0%	244	56.7%
Master’s degree (e.g., M.C.S, MA, MSc, PGCE)	78	27.5%	37	30.8%	3	14.3%	1	20.0%	112	26%
Doctorate degree (PhD)	40	14.1%	18	15.0%	4	19.1%	4	60.0%	72	16.8%
Current year of study										
First year	125	44.0%	42	35.0%	9	42.9%	1	20.0%	177	41.2%
Second year	61	21.5%	37	30.8%	6	28.6%	3	60.0%	107	24.9%
Third year	65	22.9%	31	25.8%	4	19.0%			100	23.3%
Fourth year	27	9.5%	9	7.5%	2	9.5%	1	20.0%	39	9.1%
Fifth years and above	6	2.1%	1	0.8%					7	1.6%
Generational status [First generation has been considered in this research as students who are the first in their family (foster parents, care workers, brother or sister, biological parents (if they are adopted)), or a parent with whom they have had no contact] to attend a higher education institution (Pascarella et al., 2004).										
Yes	79	27.8%	34	28.3%	7	33.3%			120	27.9%
No	193	68.0%	83	69.2%	11	52.4%	5	100.0%	292	67.9%
Prefer not to say	9	3.2%	3	2.5%	1	4.8%			13	3.0%
Other	3	1.1%			2	9.5%			5	1.2%
Home and international student status										
I am a home student *(Home students are those living in the UK or Republic of Ireland without any immigration restriction)*	138	48.6%	65	54.2%	14	66.7%	2	40.0%	219	50.9%
I am an International student	143	50.4%	55	45.8%	7	33.3%	3	60.0%	208	48.4%
Prefer not to say	3	1.1%							3	0.7%
How long have you been enrolled at your university?										
0–1 year	37	13.0%	12	10.0%	1	4.8%	1	20.0%	51	11.9%
1–2 years	95	33.5%	32	26.7%	4	19.0%	1	20.0%	132	30.7%
2–3 years	51	18.0%	31	25.8%	10	47.6%	2	40.0%	94	21.9%
3–4 years	64	22.5%	28	23.3%	1	4.8%			93	21.6%
4–5 years	28	9.9%	15	12.5%	2	9.5%	1	20.0%	46	10.7%
5 and more years	9	3.2%	2	1.7%	3	14.3%			14	3.3%

### 2.3 Ethical considerations

All study procedures were approved by the School of Education Ethics Committee on October 18th, 2022, before data collection began. Ethical guidelines were rigorously followed throughout the research to protect participants’ rights and confidentiality. Informed consent was obtained from all participants before starting the survey, and measures were implemented to safeguard their anonymity and privacy. Any potential risks or discomforts associated with participation were carefully addressed, and participants were assured of their right to withdraw from the study at any time without consequences. The researcher created the consent, debrief, privacy, and participant information documents to gain transparent and fully informed consent. To ensure that participants were informed correctly, the participant information was explained in transparent and robust language as to how data would be shared and processed.

### 2.4 Instruments

All data for this study were collected using a questionnaire administered through the JISC Online Survey. To achieve the study objectives, structured questionnaires were used, consisting of demographic characteristics and six factors: collegiate community belonging, COVID-19 student stress, degree department belonging, loneliness, coping self-efficacy, and academic anxiety. The following sections provide details on each of the questionnaires used in this study.

#### 2.4.1 Collegiate community belonging scale

The University Belonging Scale was initially developed by Slaten et al. (2018) to measure students’ university belonging. University affiliation (12 items) and Faculty and staff relations (4 items) items have been included in this study. The researcher modified the University Belonging Questionnaire to measure students’ sense of collegiate community belonging. The scale was modified to cover undergraduate and postgraduate students. These modified items were included in this study [e.g., “I have university-branded material that others can see (pens, notebooks, bumper sticker, etc.)”] was changed to “I have college-branded material that others can see (pens, notebooks, bumper sticker, etc.).” The adapted scale examined students’ general sense of collegiate community belonging (16 items, *α* = 0.94, see Appendix 1). The proposed three-factor model demonstrated a robust fit with the data, χ2(249) = 16, *p* < 0.001, CFI = 0.93, RMSEA = 0.05 (90% CI: 0.04, 0.06), SRMR = 0.05, TLI = 0.92. The standardised loadings ranged from 0.49 to 0.80 for university affiliation, 0.52 to 0.76 for university support and acceptance, and 0.78 to 0.82 for relationships with faculty and staff. Participants rated items on a 5-point Likert-type scale ranging from “Strongly disagree” to “Strongly agree,” and scores were created by taking the mean of all items.

#### 2.4.2 Degree department belonging scale

Viola and McCrone (2019) adapted the Harvard-Panorama Student Perception Survey scale on Sense of Belonging (Gehlbach, 2015) and Yorke’s (2016) sense of belonging in higher education scale according to best practices in questionnaire design. The scale was modified to cover undergraduate and postgraduate students, and these modified items were included in this study (e.g., “How well do people at … understand you as a person?”) was changed to “(How well do people at your degree department understand you as a person?).”

#### 2.4.3 COVID-19 Student Stress Questionnaire

The COVID-19 Student Stress Questionnaire (CSSQ) (Zurlo et al., 2020) is utilised to assess stress levels associated with the COVID-19 pandemic in university students. It consists of seven items that evaluate different stress sources. The CSSQ comprises three subscales that gauge stressors related to Relationships and Academic Life, Isolation, and Fear of Contagion. The questionnaire displayed strong internal consistency, with a Cronbach’s alpha value of 0.71 and McDonald’s omega of 0.71. The three-factor model (χ2 = 4.52, *p* = 0.79) demonstrated an acceptable fit across all indices (χ*2*/df ratio = 0.56; CFI = 0.95; TLI = 0.95; RMSEA = 0.06). Only the Relationships and Academic Life subscale, consisting of four items, was employed in this study. The researcher adjusted the questions in Zurlo et al. (2020) questionnaire to gain insights into how students perceived their relationships with the college and their respective departments during the post-pandemic period. For instance, the original question, “How do you perceive the relationships with your university colleagues during this period of the COVID-19 pandemic?” was rephrased to: “How have you perceived your relationships with your colleagues during the period following the COVID-19 pandemic?” and “How have you perceived your relationships with your college peers during the period following the Covid-19 pandemic?”.

#### 2.4.4 Loneliness Scale

The ULS-8, a shortened version of the revised UCLA Loneliness Scale of Hays and DiMatteo (1987), consists of 8 selected items. These items are: (a) I lack companionship, (b) There is no one I can turn to, (c) I am an outgoing person, (d) I feel left out, (e) I feel isolated from others, (f) I can find companionship when I want it, (g) I am unhappy being so withdrawn, and (h) People are around me but not with me. The study implemented a 4-point Likert scale, ranging from “never” to “always.” The ULS-8 exhibited an internal reliability of 0.84, with factor loadings between 0.71 and 0.83. Compared to the ULS-8, the ULS-6, which excluded two reverse-scored items, displayed more robust psychometric properties regarding construct validity and internal consistency. The study’s results also supported the convergent and concurrent validity of the ULS-6. Furthermore, the ULS-6 demonstrated an overall Cronbach’s alpha of 0.878 and a test–retest reliability coefficient of 0.663.

#### 2.4.5 Academic Anxiety Scale

Academic anxiety is defined as anxiety experienced in response to academic demands and the academic environment (Cassady, 2010) and is measured in this study with the 11-item Academic Anxiety Scale. The Academic Anxiety Scale uses a 4-point Likert-type response format typical to anxiety measures, with response options ranging from not at all typical of me to very typical of me (see Appendix 1). Factor loadings ranged from 0.35 to 0.79, indicating that all items significantly contributed to the measurement of academic anxiety. Reliability was confirmed with a Cronbach’s alpha of 0.89 and composite reliability of 0.91.

#### 2.4.6 The coping self-efficacy scale—short form

This questionnaire consists of 13 items that assess an individual’s perceived self-efficacy in dealing with challenges and threats. Based on self-efficacy theory, it is designed to measure changes in a person’s confidence in their ability to cope effectively (Bandura, 1997; Chesney et al., 2006). Respondents were asked to rate their confidence in performing important behaviours for adaptive coping using a 13-point scale when faced with difficulties or problems. This assessment generates three subscale scores: “problem-focused coping” (*α* = 0.91), “control of unpleasant emotions and thoughts” (α = 0.91), and “seeking support” (α = 0.80). The scale ranges from 0 (“cannot do at all”) to 5 (“moderately certain can do”) to 10 (“certain can do”). The results indicated satisfactory model fit (χ2 (62) = 152.36, *p* < 0.0001, CFI = 0.95, RMSEA = 0.08, SRMR = 0.05) (Chesney et al., 2006).

### 2.5 Data analysis

This study analysed statistics using IBM SPSS version 29 and IBM AMOS 28 software. The initial phase of the analysis centred on calculating descriptive statistics to comprehensively profile the research participants’ demographics and characteristics using SPSS version 29. In addition, AMOS 28 software was used for other research-related analyses. This foundational step was essential for delineating age, gender, education level, and other pertinent socio-demographic factors. Following the accumulation of descriptive statistics, CFA was employed to evaluate the psychometric properties of the constructs under examination. The primary objective was to ascertain how the data conformed to a pre-defined factor structure, ensuring that the observed variables adequately represented the theoretical constructs. Subsequently, each construct’s unidimensionality measures were assessed via correlation analyses, which were further validated through confirmatory assessments to analyse the interrelationships among the variables. Frequency, reliability, and correlational analyses were carried out through SPSS. Before analysis, missing responses and outliers were screened for. The first criterion was that each construct’s square root of the Average Variance Extracted (AVE) should be larger than the inter-construct correlation. The second criterion was achieved when the loading of an item for a construct was more significant than its loading for any other construct in the model. AVE was evaluated with a recommended minimum value of 0.5 for each factor (Ab Hamid et al., 2017). The item reliability of each measure was assessed through factor loading, with a value of 0.70 or higher recommended (Raubenheimer, 2004). The composite reliability of each construct was assessed using an alpha coefficient of 0.70 or higher, as suggested by Hair et al. (2020) to reflect adequate reliability.

The hypothesised theoretical model was then rigorously analysed using SEM, which facilitated a thorough investigation of the relationships among variables while accounting for measurement error and providing robust estimates of the effects among constructs. Fit indices were utilised to measure model fit. Three categories of fit indices were used: absolute fit indices, parsimony indices, and comparative indices. Absolute fit indices measure how well the proposed model reproduces the observed data, while parsimonious indices consider the model’s complexity (Marsh et al., 2013; Smith and McMillan, 2001). The most common fit index is the model chi-square (χ2). The following categories of fit indices are the parsimonious indices, which are similar to the absolute fit indices except that they consider the model’s complexity. An example is the root mean square error of approximation (RMSEA). Comparative fit indices evaluate model fit relative to an alternative baseline model (Bentler, 1990; Van Laar and Braeken, 2021). Comparative fit indices include the Tucker-Lewis (TLI) and comparative fit (CFI). The hypothesis outlined in the research model was tested, and path coefficients were calculated to determine significantly related constructs. This analysis contributed to a more nuanced understanding of how different factors interplay and shape the overarching framework under investigation. In examining the influence of socio-demographic backgrounds on the variables, a MGSEM was implemented. This analysis adhered to several critical steps specified by Lv et al. (2022) and Putnick and Bornstein (2016). Initially, the invariance of the structural equation models across demographic groups was assessed to ascertain the model’s consistency across different contexts. A primary indicator of factorial invariance was the non-significance of the difference in chi-square values between the unconstrained model (with parameters allowed to vary freely) and the measurement-weighted model (with specific parameters constrained). This assessment allowed the researcher to determine whether the constructs functioned uniformly across groups. Following this, the difference in chi-square values between the measurement-weighted and structure-weighted models was examined to evaluate metric invariance, identifying any significant disparities among groups regarding the relationships between constructs.

## 3 Results

### 3.1 CFA measurement model

A CFA was utilised to ascertain the construct validity of the latent variables and to determine the extent to which the data conforms to a pre-established measurement model. A CFA was conducted for each scale separately. A minimum alpha reliability of 0.70 is recommended to indicate adequate reliability at the construct level (Frost et al., 2007). In this study, all factors exhibited Cronbach’s *α* coefficients exceeding 0.70, indicating satisfactory reliability for all the variables under investigation. The findings indicated a reliability coefficient of 0.96 for the collegiate community belonging scale, 0.93 for the COVID-19 student stress scale, 0.94 for the degree department belonging scale, 0.91 for loneliness, 0.90 for coping self-efficacy, and 0.93 for the academic anxiety scale, all at commendable levels (see Table 2).

**Table 2:** Descriptive statistics, Cronbach’s alpha (α), and skewness and kurtosis values across study variables.

Variables	Number of items included	Cronbach’s α	Skewness	Kurtosis
Collegiate community belonging (CCB)	17	0.96	−0.07	−0.76
Degree department belonging (DB)	10	0.94	−0.09	−0.69
Loneliness	8	0.91	0.20	0.40
Academic anxiety (AA)	11	0.93	0.23	−0.92
Covid-19 student stress (CSSQ)	5	0.93	0.49	−0.78
Coping self-efficacy (CSE)	11	0.90	0.14	−0.45

In the context of employing structural equations, verifying the factors’ validity is imperative. The amalgamated survey yielded a KMO value of 0.940, signifying the suitability of the data for factor analysis. Furthermore, the Bartlett index for all variables and their dimensions was below 0.01, indicating robust correlations within the dataset. An analysis of normality revealed that the data in this study adhered to a normal distribution, as demonstrated by skewness and kurtosis values falling within the −2 to +2 and −7 to +7 ranges, respectively (Cain et al., 2017; Hair et al., 2010). The researcher conducted an assessment of standardised factor loadings and residuals and excluded items with factor loadings below 0.40 and significant standardised residuals from the model (Jackson et al., 2009). The examination of the measurement model indicated that a refined model comprising 60 items and six subscales— collegiate community belonging, degree department belonging, loneliness, COVID-19 student stress, coping self-efficacy, and academic anxiety—exhibited a satisfactory fit. Model fit was assessed using several criteria: the Satorra–Bentler scaled statistic, comparative fit index (CFI; Bentler, 1990), standardised root means square residual (SRMR), and root mean square error of approximation (RMSEA; Steiger, 2000) with a 90% confidence interval (CI). Initial examination of students’ responses showed that the data were multivariately kurtotic; therefore, robust statistics were used for all analyses. The Satorra–Bentler scaled statistic (S–B χ2) was employed because it corrects the test statistics and standard errors when data are non-normally distributed. This study utilised the two-index strategy proposed by Hu and Bentler (1999). Specifically, a desired value of less than 0.08 was sought for the SRMR. Additionally, lower values of RMSEA indicated better fit: values below 0.05 indicated good fit, values up to 0.08 indicated acceptable fit, and values exceeding 0.10 indicated poor fit (Kim et al., 2016). In reporting evidence of invariance, two criteria must be satisfied. The first criterion is that the multi-group model demonstrates adequate fit to the data. CFI and TLI values, which range from 0 to 1, indicated acceptable to excellent fit when surpassing 0.90 and 0.95, respectively (Bentler, 1990). Furthermore, Lagrange multiplier test modification indices were examined to identify untenable equality constraints. The fit indices for the confirmatory factor analysis of the full measurement model were relatively good fit (Δχ2/df = 1.9 (<3 good), RMSEA = 0.04 (<0.08 good), CFI = 0.95(>0.95 great), NFI = 0.92, TLI = 0.93, IFI = 0.93). Table 3 presents the reliability analysis results of each construct and the indicator loadings (>0.50).

**Table 3:** Factor loading, AVE, and CR of latent variables.

Construct	Item	Outer loadings	Average variance extracted (AVE)	Composite reliability (CR)
Collegiate community belonging (CCB)				
	CCB1	0.66	0.63	0.96
	CCB2	0.86		
	CCB3	0.83		
	CCB4	0.87		
	CCB5	0.77		
	CCB6	0.87		
	CCB7	0.66		
	CCB8	0.78		
	CCB9	0.82		
	CCB10	0.88		
	CCB11	0.90		
	CCB12	0.86		
	CCB13	0.70		
	CCB14	0.70		
	CCB15	0.72		
	CCB16	0.78		
Department belonging (DB)				
	DB1	0.82	0.65	0.95
	DB2	0.80		
	DB3	0.84		
	DB4	0.63		
	DB5	0.77		
	DB6	0.79		
	DB7	0.83		
	DB8	0.81		
	DB9	0.87		
	DB10	0.88		
Loneliness (LN)				
	LN1	0.69	0.57	0.90
	LN2	0.73		
	LN3	0.76		
	LN4	0.77		
	LN5	0.78		
	LN6	0.80		
Academic anxiety (AA)				
	AA1	0.77	0.59	0.94
	AA2	0.71		
	AA3	0.80		
	AA4	0.81		
	AA5	0.81		
	AA6	0.72		
	AA7	0.76		
	AA8	0.80		
	AA9	0.74		
	AA10	0.78		
	AA11	0.82		
COVID-19 student stress (CSSQ)				
	CSSQ1	0.91	0.78	0.95
	CSSQ2	0.89		
	CSSQ3	0.88		
	CSSQ4	0.88		
	CSSQ5	0.84		
Coping self-efficacy (CSE)				
	CSE1	0.85	0.63	0.95
	CSE2	0.81		
	CSE3	0.91		
	CSE4	0.92		
	CSE5	0.92		
	CSE6	0.81		
	CSE7	0.86		
	CSE8	0.84		

CSSQ, COVID-19 pandemic related student stress; DB, degree department belonging; CSE, coping self-efficacy; LN, loneliness; CCB, collegiate community belonging; AA, academic anxiety. Average variance extracted (AVE) is computed by ∑(λ2)/n; Composite reliability (CR) is computed by (∑λ)2 /(∑λ)2 + ∑ (1 – λ2), where λ = factor loadings and *n* = number of indicators of the construct.

### 3.2 Correlation analysis for the CFA measurement model

The correlation matrix highlights significant relationships among key psychological and academic variables, providing insight into students’ well-being and experiences in higher education contexts (see Table 4). Collegiate community belonging (M = 3.04, SD = 1.04) exhibited a moderate positive correlation with department belonging (*r* = 0.22, *p* < 0.01) and coping self-efficacy (*r* = 0.27, *p* < 0.01). Conversely, collegiate community belonging was negatively associated with loneliness (*r* = −0.34, *p* < 0.01) and COVID-19 student stress (*r* = −0.19, *p* < 0.01). Department belonging (M = 3.23, SD = 0.93) also demonstrated significant negative correlations with loneliness (*r* = −0.40, *p* < 0.01), academic anxiety (*r* = −0.41, *p* < 0.01), and COVID-19 student stress (*r* = −0.33, *p* < 0.01), while being positively associated with coping self-efficacy (*r* = 0.36, *p* < 0.01). Loneliness (M = 2.83, SD = 0.63) was positively correlated with academic anxiety (*r* = 0.45, *p* < 0.01) and COVID-19 student stress (*r* = 0.29, *p* < 0.01), and negatively with coping self-efficacy (*r* = −0.46, *p* < 0.01). Similarly, academic anxiety (M = 2.65, SD = 1.06) was positively correlated with COVID-19 Student Stress (*r* = 0.38, *p* < 0.01) and negatively with coping self-efficacy (*r* = −0.55, *p* < 0.01). Finally, COVID-19 student stress (M = 2.38, SD = 1.15) demonstrated a significant negative correlation with coping self-efficacy (*r* = −0.28, *p* < 0.01).

**Table 4:** Correlation between variables included in the study.

Variables	Mean	Std. deviation	1	2	3	4	5	6
Collegiate community belonging	3.039	1.037	1	0.22**	−0.34**	−0.09	−0.19**	0.27**
Department belonging	3.225	0.925		1	−0.40**	−0.41**	−0.33**	0.36**
Loneliness	2.829	0.629			1	0.45**	0.29**	−0.46**
Academic anxiety	2.649	1.062				1	0.38**	−0.55**
COVID-19 student stress	2.376	1.155					1	−0.28^**^
Coping self-efficacy	6.206	2.151						1

* *p* < 0.05; ** *p* < 0.01; *** *p* < 0.001.

### 3.3 Assessment of the measurement model

In alignment with the methodological framework set forth by Fornell and Larcker (1981), and further supported by contemporary literature (Hair et al., 2016). The AVE values, detailed in Table 3, were observed to exceed the standard threshold of 0.5 (Fornell and Larcker, 1981; Hair et al., 2016) across all constructs in the measurement model, ranging between 0.573 and 0.775, indicating strong construct validity. An AVE exceeding 0.5 indicates that the latent construct can account for more than 50% of the variance in the corresponding indicators (Hair et al., 2016). The internal consistency reliability was also examined, defined as the degree to which all items within a specific subscale measure the same concept (Green and Yang, 2015), through composite reliability metrics. This study adhered to the established threshold range of 0.70–0.95, as values below or above this threshold range raise concerns about the reliability of the measurement constructs. The findings of this study confirm that the composite reliability (CR) scores for the scales were acceptable. Notably, the Composite Reliability (CR) for collegiate community belonging were 0.96, slightly exceeding the commonly recommended threshold of 0.95, indicating potential redundancy among the items. However, all items were retained to capture the theoretical breadth of the construct. The results of the AVE and CR analyses indicate that the measurement model employed in this study is both reliable and appropriate for subsequent analytical procedures, thereby reinforcing the validity of the research framework.

### 3.4 Discriminant validity

Table 5 presents the correlations between the latent variables. Discriminant validity was evaluated by computing the square roots of the AVEs and comparing the correlation matrix’s diagonal elements with the off-diagonal elements in the corresponding rows and columns (Fornell and Larcker, 1981). The results show that the square root of AVE (diagonal bolded values) is higher than the inter-construct correlations, indicating that discriminant validity issue was not existed in this study.

**Table 5:** Discriminant validity of Fornell-Larcker criterion.

Variables	1	2	3	4	5	6
Collegiate community belonging	**0.80**					
Department belonging	0.22	**0.81**				
Loneliness	−0.34	−0.40	**0.76**			
Academic anxiety	−0.09	−0.41	0.45	**0.77**		
COVID19 student stress	−0.19	−0.33	0.29	0.38	**0.88**	
Coping self-efficacy	0.27	0.36	−0.46	−0.55	−0.28	**0.86**

The bolded values represent the square root of AVE. Other values represent intercorrelations between constructs for measuring the Fornell-Larcker criterion.

### 3.5 Test of the structural model and hypotheses

This study used AMOS 29.0 to analyse the hypothesised theoretical model using SEM. The goal of SEM in this study was to confirm the adequacy of the structural model. To assess how well the hypothesised model fits the data, the researcher calculated global fit indices. These indices indicated that the structural model fit the data well.

The fit indices for the structural model showed a strong fit, indicating a high level of fit. The specific fit indices were as follows: Δχ2/df = 1.8 (<3 is good), RMSEA = 0.04 (<0.08 is good), RMR = 0.011 (<0.05 is great), CFI = 0.94 (>0.95 is great), NFI = 0.997, TLI = 0.93, IFI = 0.93.

**Figure 2:**
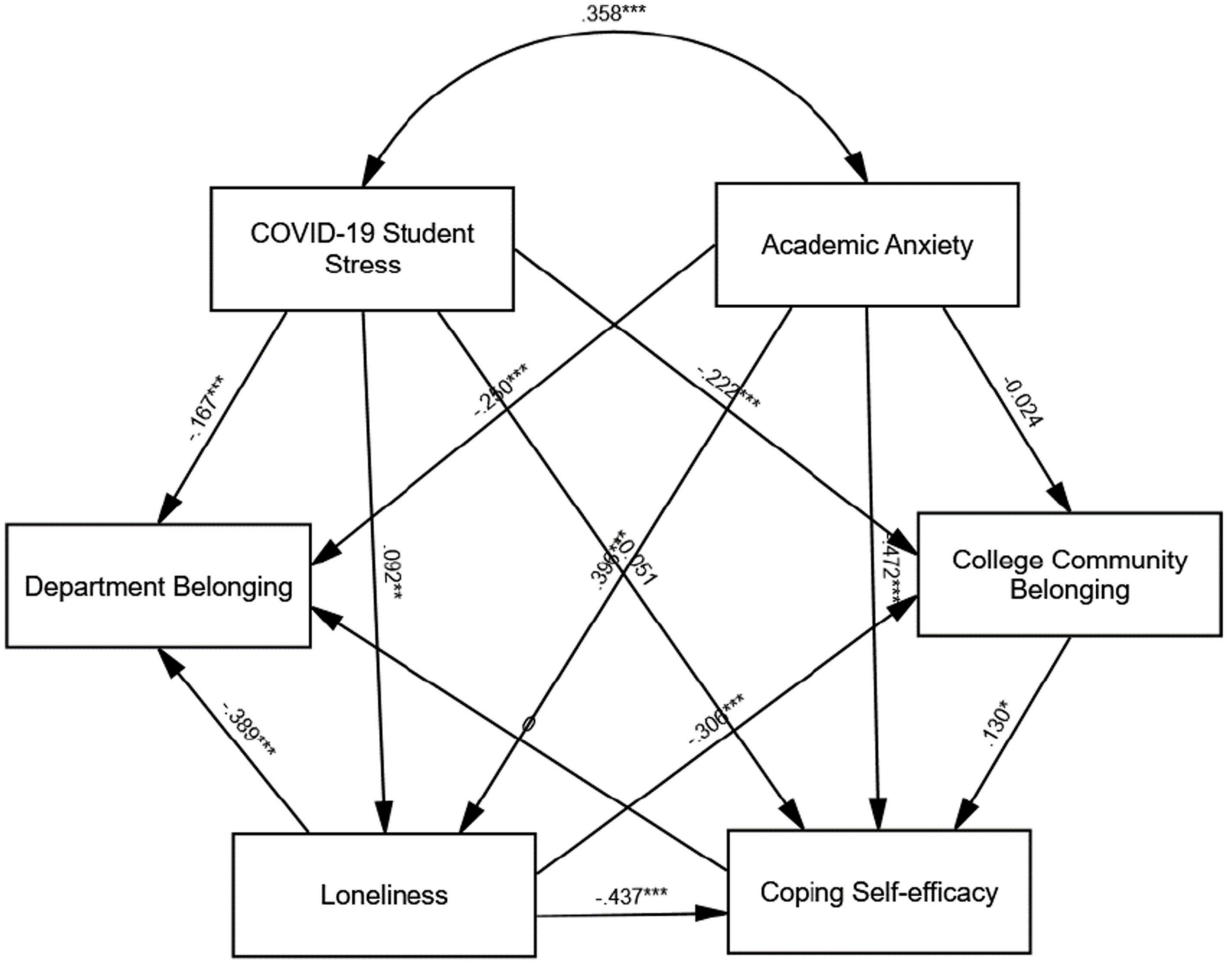
The results of the SEM statistical model for path analysis.

The results of the Structural Equation Modelling (SEM) analysis corroborated most of the proposed hypotheses, illuminating the intricate relationships among COVID-19-related stress (CSSQ), degree of belonging (DB), coping self-efficacy (CSE), loneliness (LN), collegiate community belonging (CCB), and academic anxiety (AA) (see Table 6). CSSQ exhibited a significant negative effect on DB (*β* = −0.17, *p* < 0.001), LN (*β* = 0.09, *p* < 0.01), and CCB (*β* = −0.22, *p* < 0.001), along with a noteworthy positive effect on AA (*β* = 0.36, *p* < 0.001), thereby supporting hypotheses H1, H3, H4, and H5. Conversely, the influence of CSSQ on CSE (*β* = −0.05, *p* > 0.05) was found to be insignificant, resulting in the rejection of hypothesis H2. Additionally, loneliness adversely impacted DB (*β* = −0.39, *p* < 0.001) and CCB (*β* = −0.31, *p* < 0.001), thus supporting hypotheses H6 and H7. Academic anxiety did not demonstrate a significant effect on CCB (*β* = −0.02, *p* > 0.05), leading to the rejection of hypothesis H8; however, it negatively influenced DB (*β* = −0.25, *p* < 0.001) and CSE (*β* = −0.47, *p* < 0.001), supporting hypotheses H10 and H11. Furthermore, collegiate community belonging positively predicted coping self-efficacy (*β* = 0.13, *p* < 0.05), confirming hypothesis H9, while loneliness exerted a significant negative effect on coping self-efficacy (*β* = −0.44, *p* < 0.001), thereby supporting hypothesis H12. Finally, academic anxiety emerged as a strong predictor of loneliness (*β* = 0.40, *p* < 0.001), corroborating hypothesis H14. However, coping self-efficacy did not significantly predict degree of belonging (*β* = 0.00, *p* > 0.99), resulting in the rejection of hypothesis H13. These findings underscore the pivotal role of COVID-19-related stress in influencing academic and social belonging, mediated by anxiety, loneliness, and coping self-efficacy, while also delineating distinct pathways that shape students’ experiences in higher education. The results of the SEM statistical model for path analysis are shown in Figure 2.

**Table 6:** Direct effects of structural models.

Hypotheses	Direct effect	Β	S. E	*t*-value	Results
H1	DB < --- CSSQ	−0.17***	0.04	−3.40	Supported
H2	CSE < --- CSSQ	−0.05	0.05	−0.90	Rejected
H3	LN < --- CSSQ	0.09**	0.03	2.59	Supported
H4	CCB < --- CSSQ	−0.22***	0.06	−3.68	Supported
H5	AA<--- CSSQ	0.36***	0.04	7.99	Supported
H6	DB < --- LN	−0.39***	0.07	−4.94	Supported
H7	CCB < --- LN	−0.31***	0.04	−6.48	Supported
H8	CSE < --- LN	−0.44***	0.09	−4.56	Supported
H9	CSE < --- CB	0.13*	0.06	2.02	Supported
H10	DB < --- CSE	0.00	0.04	−0.01	Rejected
H11	CCB < --- AA	−0.02	0.06	−0.34	Rejected
H12	DB < --- AA	−0.25***	0.06	−3.79	Supported
H13	CSE < --- AA	−0.47***	0.08	−5.56	Supported
H14	LN < --- AA	0.40***	0.04	8.38	Supported

CSSQ, COVID-19 pandemic related student stress; DB, degree department belonging; CSE, coping self-efficacy; LN, loneliness; CCB, collegiate community belonging; AA, academic anxiety. * *p* < 0.05; ** *p* < 0.01; *** *p* < 0.001.

## 4 Multi-group analysis

Before conducting a multi-group analysis to compare the path coefficients across gender, education level, and international and home student status, the measurement invariance of composite models (MICOM) approach (Henseler et al., 2016) was used to assess the configural, compositional, and scalar invariances (equality of means and variances). The MICOM results (Figure 2) indicated that configural and compositional invariance were established for female and male student groups, but scalar invariance was not, demonstrating partial measurement invariance. According to Henseler et al. (2016), partial measurement invariance is sufficient to conduct multi-group analyses. Subsequently, a permutation-based multi-group analysis was carried out to compare three groups (male and female students) regarding the linkages within the model.

### 4.1 Model 1. Structural equation model multi-group analysis for gender

Model 1 and the multi-group analysis results for males and females are shown in Table 7. The fit indices for the structural model indicated a good fit. The specific fit indices were as follows; (Δχ2/df = 1.67 (<3 good), RMSEA = 0.04 (<0.08 good), CFI = 0.90 (>0.85 good), TLI = 0.90, IFI = 0.91).

**Table 7:** Structural equation model multi-group analysis for gender.

			Female			Male		
Path			Estimate	Standard error	Critical ratio	Estimate	Standard error	Critical ratio
DB	<−--	CSSQ	−0.11	0.05	−1.85	−0.30**	0.09	−3.13
CSE	<−--	CSSQ	−0.07	0.06	−0.96	−0.02	0.09	−0.24
LN	<−--	CSSQ	0.07	0.04	1.64	0.15*	0.06	2.25
CCB	<−--	CSSQ	−0.24**	0.07	−3.07	−0.14	0.09	−1.39
AA	<−--	CSSQ	0.50***	0.07	6.33	0.25*	0.11	2.20
DB	<−--	LN	−0.33***	0.09	−3.69	−0.40**	0.15	−2.60
CCB	<−--	LN	−0.27***	0.05	−4.75	−0.45***	0.10	−4.49
CSE	<−--	LN	−0.40***	0.11	−3.63	−0.46**	0.17	−2.67
CSE	<−--	CCB	0.09	0.07	1.20	0.20	0.10	1.89
DB	<−--	CSE	−0.06	0.06	−0.97	0.08	0.10	0.78
CCB	<−--	AA	−0.10	0.09	−1.07	0.10	0.11	0.85
DB	<−--	AA	−0.38***	0.08	−4.34	−0.16	0.13	−1.22
CSE	<−--	AA	−0.44***	0.10	−4.16	−0.35*	0.14	−2.42
LN	<−--	AA	0.38***	0.06	6.13	0.47***	0.09	5.14

* *p* < 0.050, ** *p* < 0.010, *** *p* < 0.001. CSSQ, COVID-19-related student stress; CSE, coping self-efficacy; DB, department belonging; LN, loneliness; CCB, collegiate community belonging; AA, academic anxiety.

The multi-group structural equation modelling analysis conducted to compare female and male students demonstrated notable gender-based differences in the interactions related to COVID-19-related stress, specifically with regard to the constructs CSSQ, CSE, DB, LN, CCB, and AA (see Table 7). The results indicated that CSSQ significantly diminished CCB for female students (*β* = −0.24, *p* < 0.01), while no significant effect was observed for male students (*β* = −0.14, *p* > 0.05). Additionally, CSSQ significantly increased AA for both female and male students; however, the effect was more pronounced among females (*β* = 0.50, *p* < 0.001) compared to males (*β* = 0.250, *p* < 0.05). Furthermore, CSSQ had a positive influence on LN for male students (*β* = 0.15, *p* < 0.05), but not for female students (*β* = 0.07, *p* = 0.10). Loneliness had a deleterious effect on both DB and CCB across both groups, with male students exhibiting a more substantial impact on CCB (*β* = −0.45, *p* < 0.001) compared to their female counterparts (*β* = −0.27, *p* < 0.001). Similarly, LN adversely affected DB more significantly for males (*β* = −0.40, *p* < 0.01) than for females (*β* = −0.33, *p* < 0.001). Academic anxiety was found to significantly contribute to LN in both demographics, with males displaying a greater effect (*β* = 0.47, *p* < 0.001) than females (*β* = 0.38, *p* < 0.001). CSE was negatively impacted by LN across both genders, with male students experiencing a stronger effect (*β* = −0.46, *p* < 0.01) than female students (*β* = −0.40, *p* < 0.001). Similarly, AA negatively influenced CSE in both groups, yielding a stronger effect for females (*β* = −0.44, *p* < 0.001) compared to males (*β* = −0.345, *p* < 0.05).

### 4.2 Model 2. Structural equation model multi-group analysis for international and home students

Table 8 displays the Model 2, multi-group analysis results for home and international students. The fit indices for the structural model showed a good fit. The specific fit indices were as follows; (Δχ2/df = 1.6 (<3 good), RMSEA = 0.03 (<0.08 good), CFI = 0.92 (>0.95 great), TLI = 0.90, IFI = 0.92).

**Table 8:** Structural equation model multi-group analysis for international and home students’ status.

			International			Home		
Path			Estimate	Standard error	Critical ratio	Estimate	Standard error	Critical ratio
DB	<−--	CSSQ	−0.11	0.06	−1.68	−0.21**	0.07	−2.98
CSE	<−--	CSSQ	−0.00	0.09	−0.02	−0.05	0.07	−0.68
LN	<−--	CSSQ	0.07	0.05	1.25	0.10*	0.04	2.11
CCB	<−--	CSSQ	−0.27**	0.09	−3.02	−0.16*	0.08	−1.98
AA	<−--	CSSQ	0.43***	0.09	4.82	0.45***	0.08	5.03
DB	<−--	LN	−0.55***	0.10	−5.40	−0.168	0.11	−1.44
CCB	<−--	LN	−0.26***	0.06	−3.99	−0.35***	0.06	−5.04
CSE	<−--	LN	−0.33*	0.13	−2.43	−0.55***	0.13	−4.00
CSE	<−--	CCB	0.22*	0.09	2.23	0.04	0.08	0.44
DB	<−--	CSE	0.00	0.07	0.07	−0.04	0.07	−0.51
CCB	<−--	AA	−0.10	0.09	−1.10	0.07	0.10	0.68
DB	<−--	AA	−0.16	0.08	−1.87	−0.31**	0.10	−3.07
CSE	<−--	AA	−0.41***	0.12	−3.32	−0.54***	0.12	−4.46
LN	<−--	AA	0.45***	0.06	6.73	0.35***	0.06	5.16

* *p* < 0.050, ** *p* < 0.010, *** *p* < 0.001. CSSQ, COVID-19-related student stress; CSE, coping self-efficacy; DB, department belonging; LN, loneliness; CCB, collegiate community belonging; AA, academic anxiety.

The multi-group structural equation modelling (SEM) analysis comparing international and domestic students revealed nuanced differences in the interactions among the constructs of CSSQ, CSE, DB, LN, CCB, and AA across the two student groups (see Table 8). CSSQ significantly mitigated CCB for both groups; however, its effect was more pronounced among international students (*β* = −0.27, *p* < 0.01) compared to home students (*β* = −0.16, *p* < 0.05). Additionally, CSSQ exhibited a significant positive impact on AA for both groups (*β* = 0.43, *p* < 0.001 for international students; *β* = 0.45, *p* < 0.001 for home students). Notably, only home students experienced a statistically significant increase in LN attributable to CSSQ (*β* = 0.10, *p* < 0.05). Moreover, LN negatively influenced CCB in both groups, with a stronger effect for home students (*β* = −0.35, *p* < 0.001) in contrast to international students (*β* = −0.26, *p* < 0.001). Furthermore, LN exhibited a notable reduction in DB for international students (*β* = −0.55, *p* < 0.001), whereas the effect was not statistically significant for home students (*β* = −0.17, *p* > 0.05). AA was positively correlated with LN for both groups, exhibiting a more substantial effect in the international student cohort (*β* = 0.45, *p* < 0.001) compared to home students (*β* = 0.35, *p* < 0.001). The relationship between CSE and CCB was significant exclusively for international students (*β* = 0.22, *p* < 0.05), implying that a sense of belonging to the college plays a more critical role in enhancing this group’s confidence in their coping capabilities. In contrast, LN demonstrated a strong negative effect on CSE for both groups, with a more significant impact observed among home students (*β* = −0.55, *p* < 0.001) as opposed to international students (*β* = −0.33, *p* < 0.05). Finally, AA adversely affected CSE in both groups, with a more pronounced effect for home students (*β* = −0.54, *p* < 0.001) than for international students (*β* = −0.41, *p* < 0.001).

### 4.3 Model 3. Structural equation model multi-group analysis for educational level

Table 9 displays the Model 3, multi-group analysis results for undergraduate and postgraduate students. The fit indices for the structural model showed a strong fit, indicating a high level of fit. The specific fit indices were as follows; (Δχ2/df = 1.7 (<3 good), RMSEA = 0.04 (<0.08 good), CFI = 0.95 (>0.95 great), TLI = 0.96, IFI = 0.95).

**Table 9:** Structural equation model multi-group analysis for educational level.

			Undergraduate			Postgraduate		
Path			Estimate	Standard error	Critical ratio	Estimate	Standard error	Critical ratio
DB	<−--	CSSQ	−0.19**	0.061	−3.15	−0.10	0.083	−1.15
CSE	<−--	CSSQ	−0.08	0.070	−1.15	0.02	0.091	0.25
LN	<−--	CSSQ	0.10*	0.041	2.32	0.10	0.066	1.49
CCB	<−--	CSSQ	−0.30***	0.074	−4.01	−0.08	0.103	−0.80
AA	<−--	CSSQ	0.40***	0.084	4.69	0.45***	0.093	4.91
DB	<−--	LN	−0.29**	0.112	−2.62	−0.49***	0.109	−4.47
CCB	<−--	LN	−0.27***	0.058	−4.60	−0.36***	0.080	−4.47
CSE	<−--	LN	−0.37**	0.134	−2.76	−0.43***	0.128	−3.39
CSE	<−--	CCB	0.08	0.084	0.90	0.18	0.095	1.88
DB	<−--	CSE	−0.00	0.072	−0.03	0.02	0.078	−0.30
CCB	<−--	AA	−0.00	0.092	−0.01	−0.09	0.111	−0.77
DB	<−--	AA	−0.20*	0.090	−2.17	−0.30**	0.100	−3.03
CSE	<−--	AA	−0.57***	0.124	−4.63	−0.42***	0.122	−3.46
LN	<−--	AA	0.40***	0.062	6.49	0.39***	0.077	5.06

* *p* < 0.050, ** *p* < 0.010, *** *p* < 0.001. CSSQ, COVID-19-related student stress; CSE, coping self-efficacy; DB, department belonging; LN, loneliness; CCB, collegiate community belonging; AA, academic anxiety.

The multi-group structural equation modelling (SEM) analysis conducted to compare undergraduate and postgraduate students has illuminated significant differences in the interactions among various constructs, namely CSSQ, CSE, DB, LN, CCB, and AA, within these cohorts (see Table 9). The results indicate that CSSQ has a negative effect on DB among undergraduate students (*β* = −0.19, *p* < 0.01), whereas this effect is not statistically significant for postgraduate students (*β* = −0.10, *p* > 0.05). Furthermore, CSSQ significantly reduces CCB solely for undergraduates (*β* = −0.30, *p* < 0.001), indicating that undergraduate students exhibit heightened sensitivity to stress-related disruptions in their sense of academic and social belonging. AA is significantly and positively associated with CSSQ in both groups, with slightly higher coefficients for postgraduates (*β* = 0.45, *p* < 0.001) than undergraduates (*β* = 0.40, *p* < 0.001), underscoring the pervasive impact of stress on academic pressures. Additionally, LN is positively affected by CSSQ in undergraduates (*β* = 0.10, *p* < 0.05), a finding not observed among postgraduates (*β* = 0.10, *p* > 0.05). This result underscores the increased vulnerability of undergraduate students to stress-induced feelings of isolation. LN significantly diminishes DB (*β* = −0.29, *p* < 0.01) and CCB (*β* = −0.27, *p* < 0.001) in the undergraduate group, while displaying even more pronounced negative effects on both DB (*β* = −0.49, *p* < 0.001) and CCB (*β* = −0.36, *p* < 0.001) for postgraduate students. Moreover, AA has a significant positive impact on LN for both student groups, with slightly greater effects observed in undergraduates (*β* = 0.40, *p* < 0.001) compared to postgraduates (*β* = 0.39, *p* < 0.001). Although AA exerts a direct negative influence on DB for both undergraduates (*β* = −0.20, *p* < 0.05) and postgraduates (*β* = −0.30, *p* < 0.01), its impact on CCB does not reach statistical significance for either group. Furthermore, AA reduces CSE across both groups, with a stronger effect noted among undergraduates (*β* = −0.57, *p* < 0.001) than postgraduates (*β* = −0.42, *p* < 0.001).

## 5 Discussion and conclusion

This study utilised Structural Equation Modelling and multi-group structural equation modelling to examine the proposed relationships among collegiate community belonging, degree department belonging, loneliness, COVID-19 pandemic-related student stress, coping self-efficacy, and academic anxiety within the context of a university in the UK. Despite the recognised importance of belonging dimensions, a significant gap persists in the literature regarding the interaction between overall collegiate community belonging and belonging specific to degree departments. Furthermore, recent studies yield varied findings concerning student stress, anxiety, belonging, loneliness, and coping self-efficacy in both pandemic and post-pandemic contexts, highlighting the necessity for further exploration in this area. Consequently, this study is innovative in its concurrent analysis of the associations among collegiate community belonging, degree department belonging, loneliness, COVID-19-related student stress, coping self-efficacy, and academic anxiety within the specific student cohort. Additionally, the research sought to understand how these interconnected factors influenced students’ academic and social experiences in the post-COVID-19 pandemic while considering variables such as gender, educational level, and status as domestic or international students across undergraduate and postgraduate programs, thereby differentiating it from prior research. The results revealed significant correlations among these factors, providing insights into the complex dynamics of student experiences during this unprecedented period. According to the proposed hypothetical model, the research findings did not support hypotheses 2, 10, and 11. In addition, standardised regression coefficients (*β*) are typically classified as follows: coefficients ranging from 0.10 to 0.30 are deemed small, those between 0.30 and 0.50 are considered moderate, and values exceeding 0.50 are categorised as large (Kline, 2023). In this research, many of the identified associations fall within the moderate-to-large range, signifying notable psychological and behavioural impacts.

The analysis indicates that the relationships among the variables display robustness and significance across the study’s cohort. These findings raise critical questions regarding the universality of the challenges encountered by students in the contemporary educational landscape, suggesting that the impact of the pandemic has fostered a shared experience that transcends demographic categories. The trends observed in the data indicate that stress related to COVID-19 has adversely impacted students’ degree department belonging, particularly among male, home, and undergraduate students. The analysis reveals that the experience of loneliness has a significantly stronger negative impact on males in the department belonging (*β* = −0.40, *p* < 0.01). In contrast, females experience a relatively lesser impact (*β* = −0.33, *p* < 0.001). These findings imply that male students may experience a deeper erosion of their sense of belonging due to feelings of loneliness. This could be attributed to the possibility that males possess fewer effective social coping strategies, which may hamper their ability to manage and mitigate feelings of isolation (Hawkley and Cacioppo, 2010; Livingston et al., 2022; Mahalik et al., 2003). On the other hand, female students, despite also experiencing the detrimental effects of loneliness, appear to employ social support-seeking behaviours that help alleviate some of its adverse consequences. This proactive approach might help them maintain their sense of belonging and reduce the likelihood of engaging in dysfunctional behaviours linked to loneliness (Kleinberg et al., 2013; Tamres et al., 2002).

The analysis indicates a significant negative effect on undergraduate students (*β* = −0.19, *p* < 0.01), suggesting that they experience stress-related disruptions in their sense of belonging within their academic departments. This vulnerability may stem from their relatively weaker academic identities and a lack of well-developed coping strategies, which can limit their ability to navigate challenging situations effectively (Tinto, 1993). In contrast, the effect for postgraduates is not statistically significant (*β* = −0.10, *p* > 0.05), highlighting that this group is less affected by stress in terms of their departmental belonging. This resilience may be attributed to their enhanced academic experience, greater self-regulation skills, and more established coping mechanisms, which collectively better equip them to maintain a sense of belonging even in the presence of stressors (De la Fuente et al., 2018; Zimmerman, 2002). Thus, the findings suggest a developmental trajectory where the ability to cope with stress and foster a sense of belonging improves with increased academic maturity (Skinner and Pitzer, 2012). This decline in belonging may be linked to the disruption of traditional academic activities and the challenges associated with maintaining social connections with peers and the academic community during the pandemic (Potts, 2021). The upheaval caused by the pandemic has likely impeded students’ ability to feel integrated and supported within their chosen academic fields (Hagedorn et al., 2022). Furthermore, among male students, undergraduate students, and home students, the impact of COVID-19-related stress significantly affected feelings of loneliness. This finding emphasises the correlation between increased stress levels during the pandemic and heightened feelings of isolation among male undergraduates, potentially attributed to societal norms that discourage emotional vulnerability and impede help-seeking behaviours (Wong et al., 2021). In addition, Shin and Park (2023) underscores that men typically have less robust social support networks compared to their female counterparts, which can intensify feelings of loneliness and isolation when they are faced with stressors. These findings imply that the effects of stress on men’s mental health may be exacerbated by a propensity to retreat from social interactions.

The isolation stemming from lockdown measures and the restricted opportunities for social interaction may have exacerbated these feelings of loneliness (Ellis et al., 2020). Moreover, the adverse effects of COVID-19 pandemic related stress also notably influenced college community belonging specifically among undergraduate students, as well as international students and female students. The observed moderate negative effect for females (*β* = −0.24, *p* < 0.01) indicates that elevated levels of COVID-19-related stress have a detrimental impact on females’ sense of belonging within the college community (Thomson et al., 2023). This suggests that, as stress levels increase due to the pandemic, many female students may feel increasingly isolated or disconnected from their peers and the broader campus environment. In contrast, the non-significant effect observed for males aligns with existing research that suggests men often employ different coping mechanisms when faced with stressors. Such strategies may include focusing on problem-solving or seeking social support in ways that mitigate the adverse effects of stress on their sense of community belonging (Tamres et al., 2002). This discrepancy highlights the importance of understanding gender differences in stress responses and coping strategies, especially in the context of mental health and community engagement during challenging times. The analysis also reveals that the stress induced by the COVID-19 pandemic has had a more pronounced negative impact on the sense of community belonging among international students compared to their domestic counterparts. Specifically, the coefficient for international students indicates a strong negative effect (*β* = −0.27, *p* < 0.01), suggesting that this group experienced significantly greater challenges in maintaining their sense of belonging during the pandemic. In contrast, home students demonstrated a comparatively lesser negative effect on their community belonging (*β* = −0.16, *p* < 0.05). Many of these students encountered disruptions in their education, concerns regarding their immigration status, and isolation from their support networks (Schartner, 2023). The combination of these factors suggests a disproportionate burden on this group compared to their domestic counterparts, underscoring the need for targeted support and resources to help them navigate these unprecedented challenges. Moreover, the negative impact of stress on a student’s sense of belonging is predominantly observed among undergraduates, with a statistical coefficient of *β* = −0.30 (*p* < 0.001), signifying a strong correlation. This effect is not found to be significant for postgraduate students. This finding suggests that undergraduate students experience a pronounced sense of disconnection when faced with stress, highlighting their greater dependence on institutional support and peer networks for successful academic adjustment.

Research by Astin (1999) reinforces this notion, indicating that younger students are particularly vulnerable to feelings of isolation and require a strong community presence to navigate their educational journeys effectively (Blakemore and Mills, 2014). In contrast, postgraduate students often embody a more autonomous and goal-oriented approach to their studies (White and Ingram, 2023). They typically possess a greater capacity for self-directed learning and may engage with their academic challenges in a way that diminishes their need for communal belonging as an effective coping strategy (Kranzow and Hyland, 2016; Porter et al., 2020). Research by McPherson et al. (2018) further supports this distinction, illustrating that postgraduates are less likely to rely on their peers or institutional frameworks for emotional or academic support during stressful periods. This divergence underscores the importance of tailored support structures for undergraduates, who require more comprehensive resources to foster a sense of belonging in their educational experience.

COVID-19 related stress had a significant positive influence on academic anxiety for both female and male students across undergraduate and postgraduate levels, as well as for both home and international students. This implication suggests that pandemic related stress heightened academic anxiety across all educational levels (Brown et al., 2023; Chen and Lucock, 2022; Jackman et al., 2022). The substantial effect size observed for females (*β* = 0.50, *p* < 0.001) indicates a heightened susceptibility to academic anxiety stemming from stress. This finding aligns with previous studies, such as the work by McLean et al. (2011), which illustrate that women tend to experience more significant anxiety-related challenges. These challenges can manifest in various ways, including difficulties concentrating, increased avoidance of academic tasks, and compromised performance, ultimately impacting their overall sense of belonging and educational experience (Farhane-Medina et al., 2022; McLean et al., 2011). Additionally, both groups, comprising international students and home students, exhibited a remarkably strong and similar positive correlation between stress related to the COVID-19 pandemic and academic anxiety, with statistical analyses revealing coefficients of *β* = 0.43 (*p* < 0.001) for international students and *β* = 0.45 (*p* < 0.001) for home students. This close alignment in results indicates that the experience of academic anxiety is a widespread issue that transcends individual backgrounds and circumstances. Rather than being an isolated phenomenon related to specific student demographics, the findings underscore that academic anxiety is a universal concern affecting students regardless of their nationality or residence status.

The uncertainties and disruptions brought about by the pandemic likely intensified concerns regarding academic performance and future prospects. CSSQ had no significant impact on CSE for both males and females, home, and international students, as well as at undergraduate and postgraduate levels. The findings align with prior research that underscores the disruptive effects of pandemic-related stressors, which include the loss of routine, social isolation, and heightened academic uncertainty (Rahiem et al., 2021; Smith et al., 2020). These factors collectively detrimentally influence students’ sense of belonging (Jaremka et al., 2022). Notably, the insignificant effect of CSSQ on CSE suggests that stress does not directly diminish students’ confidence in their coping abilities. Rather, it operates through intermediary variables such as loneliness and academic anxiety. This supports earlier findings indicating that the relationship between stress and self-efficacy is frequently mediated by emotional and relational factors (Jerusalem and Schwarzer, 2014; O'Leary, 1992).

The findings of this study suggest that undergraduate students demonstrate a higher vulnerability to pandemic-related stressors, which adversely affects both their sense of department belonging and collegiate community belonging. This increased susceptibility can be attributed to their position within the academic trajectory; undergraduates typically find themselves in the initial stages of developing their academic identities and establishing social networks. Consequently, they exhibit heightened sensitivity to disruptions caused by external stressors, such as the pandemic, which may undermine their social integration and sense of community (Tinto, 1993). In contrast, postgraduate students encounter a distinct set of challenges. Although they may not experience the same level of stress as undergraduates, they frequently contend with substantial feelings of loneliness (Janta et al., 2014), which can detrimentally impact their sense of belonging within both their departments and the broader collegiate community. The inherently independent nature of postgraduate studies often exacerbates this sense of isolation, as students typically invest extended periods working autonomously on their research or projects, resulting in fewer opportunities for social interaction and engagement with peers and faculty members (King, 2022). This underscores the critical importance of fostering supportive environments that encourage connection and collaboration among postgraduate students, thereby alleviating potential negative effects of loneliness and enhancing their sense of belonging within advanced educational settings (Sverdlik et al., 2018).

Loneliness has emerged as a significant mediator within academic environments, profoundly influencing two essential components of the student experience: a sense of belonging within degree department and a sense of collegiate community. Notably, the analysis reveals a more pronounced negative impact of loneliness (LN) on the sense of belonging within the college community for males (*β* = −0.45) compared to females (*β* = −0.27) across both undergraduate and postgraduate levels. This disparity suggests that male students may experience greater difficulties in forging connections and feeling integrated within their academic environment. These patterns highlight a gender disparity in how LN affects feelings of belonging. Furthermore, when examining its effects on departmental belonging, the trend continues; males reported a more significant negative impact (*β* = −0.40) compared to females (*β* = −0.33). These results underscore the need to consider gender differences when analysing the repercussions of LN within educational environments, as males appear to be more strongly affected in both college community and departmental contexts. In addition, home students exhibited a notable correlation between COVID-19 related stress and feelings of loneliness (*β* = 0.10, *p* < 0.05). This finding suggests that as home students navigate the challenges posed by the pandemic, they may become increasingly lonely, indicating a heightened vulnerability to emotional distress in the face of stressors. In contrast, international students do not show the same relationship between stress and loneliness. This disparity may imply that international students have developed more effective coping mechanisms for dealing with loneliness, potentially stemming from prior experiences of separation from their home countries (Alshammari et al., 2023; Zheng et al., 2023). Such adaptive strategies could include strong support networks formed within their communities, familiarity with managing feelings of isolation, and a greater resilience built through navigating cultural transitions (Hendrickson et al., 2011; Lin et al., 2012). These patterns also underscore the detrimental effects that heightened feelings of isolation can have on an individual’s connection to their academic community, thereby diminishing their overall sense of belonging within their respective degree departments.

The research findings reveal a significant positive effect of stress-induced loneliness among undergraduates, evidenced by a coefficient of *β* = 0.10 (*p* < 0.05). This suggests that undergraduates, who are often navigating new academic environments and social settings, are particularly vulnerable to feelings of isolation during periods of heightened stress. This observation aligns with research conducted by McLean et al. (2023), which indicates that younger students face greater challenges in establishing social connections and coping with social isolation as they adapt to the demands of college life. In contrast, the analysis shows no statistically significant effect for postgraduates (*β* = 0.10, *p* > 0.05). This lack of significance implies that postgraduates may experience less stress-induced loneliness, likely due to their more established social and professional networks. Often, these individuals may have more time to cultivate relationships and support systems, which can serve as a buffer against the social isolation commonly felt during academic stress. Therefore, it appears that the transitional phase of undergraduate studies poses a unique challenge that is less pronounced among those in postgraduate programs. The nature of loneliness is intricately linked to social disconnection (Chen et al., 2024), which likely hinders students’ capacity to engage in meaningful interactions with their academic peers and department members. This, in turn, diminishes their sense of belonging within their educational settings. Vasileiou et al. (2019) suggest that the inability to cultivate connections can establish barriers to the formation of supportive relationships, which are vital for both academic achievement and personal well-being. Furthermore, this study revealed that loneliness adversely affects the sense of community at the college level for both male and female students, as well as across both undergraduate and postgraduate cohorts. This pervasive sense of loneliness indicates a complex, intertwined relationship wherein feelings of detachment may influence one’s overall sense of community, potentially mediated by shared experiences of challenge or adversity (Nunn et al., 2021). Such experiences can foster a collective understanding of isolation that affects the manner in which students relate to one another within the college context. These findings highlight the crucial role of social connectedness in fostering a sense of belonging and enhancing coping self-efficacy among students. The literature supports this premise, illustrating that feelings of isolation can inhibit students’ abilities to establish meaningful relationships within both academic and social environments. This disruption ultimately leads to reduced engagement and a weakening of their academic and social identities (Baumeister and Leary, 1995). Moreover, the pronounced impact of loneliness on the sense of belonging among international students warrants particular attention. This demographic frequently encounters unique vulnerabilities, navigating cultural and linguistic barriers while also facing limited access to established support networks (Baker et al., 2018; Glass and Westmont, 2014). Their experiences underscore the necessity for targeted support systems that can effectively bridge these gaps, ensuring that all students, irrespective of their backgrounds, experience a sense of belonging and community throughout their educational journeys.

The association between coping self-efficacy and loneliness was found to be significant for both females and males and at undergraduate and postgraduate levels, as well as for both home and international students. This suggests that higher coping self-efficacy is linked to reduced feelings of loneliness in students. The analysis reveals a more pronounced negative impact on coping self-efficacy among home students (*β* = −0.55) compared to their international counterparts (*β* = −0.33). This indicates that home students are likely to experience a more significant decline in their ability to cope during periods of loneliness. One possible explanation for this disparity is that home students may possess lower levels of resilience (Kamara, 2019), which hampers their ability to manage feelings of isolation effectively. In contrast, international students often develop strong self-reliance skills as a result of navigating life in a new country, which may better equip them to handle loneliness and its associated challenges. This resilience could stem from their experiences of adapting to unfamiliar environments and cultures, allowing them to maintain a more stable sense of coping self-efficacy even in difficult circumstances (Sawir et al., 2008). Individuals who believe in their ability to manage stress and overcome challenges are better at maintaining social connections and reducing experiences of isolation (Brandt et al., 2022). Students who have greater confidence in their ability to cope with challenges are better able to interact with their academic communities, fostering a stronger sense of belonging (Cena et al., 2021; Strayhorn, 2018). Sufficient confidence in coping skills enables students to actively engage with their academic communities (Skinner and Pitzer, 2012). In addition, there is a significant positive relationship between academic anxiety and loneliness, which is significant for both male students and at undergraduate and postgraduate levels, particularly among international students. This indicates that heightened academic anxiety is associated with increased feelings of loneliness (Haikalis et al., 2022). This connection emphasises the interplay between academic pressures and social well-being, especially for international students who may encounter additional stressors such as cultural adjustment and being away from family (Mesidor and Sly, 2016; Newsome and Cooper, 2016). Increased levels of academic anxiety can lead to social withdrawal and feelings of isolation among students (Cruz et al., 2023). The bidirectional relationship between academic anxiety and loneliness, where anxiety heightens feelings of isolation and loneliness exacerbates academic worries, creates a self-perpetuating cycle that undermines students’ well-being (Munir et al., 2015; Sadoughi and Hesampour, 2016). The significant reduction in coping self-efficacy attributable to academic anxiety further highlights the debilitating effect of anxiety on students’ perceived ability to manage academic demands, consistent with Bandura’s (1997) theory of self-efficacy. These findings emphasize the importance of addressing anxiety to break this cycle, with interventions such as stress management training, counselling, and peer support proving effective in reducing anxiety and enhancing self-efficacy (Richardson et al., 2012). In conclusion, this study highlighted the complex relationships between pandemic related stress, department and collegiate community belonging, loneliness, self-efficacy, and academic anxiety among undergraduate and postgraduate students during the post-COVID-19 pandemic. It emphasised the need to address COVID-19-related stress and enhance coping self-efficacy to improve students’ sense of belonging, reduce academic anxiety, and alleviate loneliness. Addressing these factors could improve students’ academic and social outcomes during challenging times. Understanding these dynamics can assist educational institutions in developing targeted interventions to support students’ well-being and academic success.

### 5.1 Implications

This study presents several significant implications for educational institutions, particularly concerning student well-being and academic performance during periods of increased stress, such as the COVID-19 pandemic. The findings highlight the necessity for targeted interventions and policy modifications aimed at better-supporting students across diverse demographic groups, including gender, educational level, and home versus international status, during and after challenging circumstances. The negative impact of COVID-19-related student stress on students’ sense of belonging—both within their specific academic departments and in the wider collegiate community —highlights a critical need for educational institutions to focus on addressing the stressors brought on by the pandemic. In the post-pandemic environment, colleges and universities must implement targeted strategies and support systems to reduce this stress. By doing so, they can foster greater student integration and ensure that all students feel connected and valued in their academic journeys. Additionally, the persistent adverse effects of the pandemic, such as loneliness and mental health challenges, warrant the development of comprehensive mental health programs and the establishment of consistent support structures to aid students in coping with the disruptions caused by the pandemic. This is especially critical for undergraduate students and home students, as they reported a significant decline in their sense of academic belonging due to pandemic-induced stress. In addition, recognising the significance of understanding the differences in stress responses and coping strategies between genders is crucial, particularly in the realm of mental health and community engagement. These differences can greatly influence how individuals experience and manage stress, especially during challenging times such as crises or uncertainties. Men and women may exhibit distinct physiological and psychological reactions to stress (Kajantie and Phillips, 2006), which can affect their ability to cope effectively. For instance, research suggests that women are more likely to engage in social coping mechanisms, seeking support from friends and family (Cholankeril et al., 2023), while men may adopt problem-focused approaches that involve more solitary or active strategies (Hidayat and Toybah, 2025). This nuanced understanding can inform mental health professionals and community leaders in tailoring interventions that resonate with diverse populations. Additionally, fostering awareness of these gender differences can enhance community engagement efforts, enabling collaborative support systems that acknowledge and address the specific needs of different groups during difficult periods. Institutions could consider offering more frequent virtual or hybrid academic and social activities to help maintain connections within academic departments and the broader collegiate community.

The significant correlation between coping self-efficacy and alleviating academic anxiety and loneliness underscores the importance of enhancing students’ coping mechanisms to promote improved mental health outcomes (Cheng, 2023; Tu and Zhang, 2015). Initiatives designed to assist students in developing resilience and managing stress related to academic and personal challenges should be incorporated into university support services (Dohaney et al., 2020; Eisenberg et al., 2016). Implementing workshops or training sessions focusing on stress management, time management, and problem-solving skills could prove particularly beneficial, especially for female and international students who may face additional pressures in these contexts. This approach can diminish anxiety levels and foster a stronger sense of belonging, cultivating a more inclusive and supportive environment (Holdsworth et al., 2018). Moreover, female students in this study have reported experiencing elevated levels of academic anxiety, which correlates with both stress and their sense of belonging within their academic departments. This indicates a pressing need for targeted academic support systems for female students, including mentoring programs, peer study groups, and initiatives specifically tailored to their academic needs (Lunsford et al., 2017; Meschitti and Lawton Smith, 2017). Male students and undergraduates exhibited a more pronounced association between pandemic-related stress and feelings of loneliness, suggesting that targeted support initiatives aimed at mitigating social isolation and fostering peer interaction would be especially advantageous for these groups. For international students, language barriers may create feelings of isolation, making it difficult to forge meaningful connections with peers and integrate into their new environment. The inability to express thoughts and ideas clearly can lead to misunderstandings and frustration, which may discourage students from engaging in conversations or participating in social activities. Additionally, social isolation can arise from being away from familiar support systems and cultural contexts, leaving international students feeling disconnected (Trusty and Chun-Kennedy, 2023). Furthermore, visa-related stress adds another layer of pressure, as the uncertainty around their legal status and future can exacerbate feelings of anxiety and loneliness (Lynch et al., 2024). These factors highlight the unique challenges faced by international students and underscore the importance of targeted support services to address their specific needs. International students, who face heightened academic anxiety and feelings of isolation, may greatly benefit from specialised support systems that address cultural adjustments, overcome language barriers, and navigate the challenges associated with being away from home (Alharbi and Smith, 2018; Lillyman and Bennett, 2014; Wang, 2018). The positive correlation between coping self-efficacy and feelings of belonging within both academic departments and the college indicates that establishing robust support networks can enhance students’ coping strategies (Nowicki, 2008; Raymond and Sheppard, 2018). These networks could include peer mentorship initiatives, faculty engagement programs, and student-led social events. For postgraduate and international students, who demonstrated a significant link between their sense of belonging and coping self-efficacy, universities could improve orientation programs, create advisory groups for international students, and provide resources to facilitate connections with peers and faculty.

The findings of this study suggest that a strong sense of belonging within an academic department can effectively reduce academic anxiety, particularly among female students. This underscores academic departments’ need to cultivate a more inclusive and supportive educational environment where students feel valued and integrated. Academic support may involve increased accessibility to faculty, regular check-ins with academic advisors, and programs that promote collaborative learning (Pham and Muralles, 2023; Renner and Skursha, 2023). Such initiatives would alleviate academic pressure and enhance students’ confidence in their academic skills. Notably, the study indicated that feelings of loneliness could paradoxically enhance students’ sense of belonging within the broader collegiate community. This observation highlights the potential for shared challenges, such as the collective experience of the pandemic, to foster a sense of unity among students. Universities have the opportunity to leverage this understanding by promoting initiatives that foster a sense of community among students, emphasizing shared challenges and collective achievements. Such initiatives can create an environment where students feel interconnected through shared experiences. Institutions may further enhance feelings of collective belonging by encouraging teamwork and facilitating open dialogue among student groups (Masika and Jones, 2016). In addition, with college belonging negatively impacted by stress, universities could create or expand peer mentoring and student networks that help students develop stronger connections to their peers and academic communities (Yomtov et al., 2017). These networks could also facilitate study groups, discussion forums, or virtual meetups to strengthen students’ sense of belonging. This research highlights the complex interrelationships among stress, belonging, loneliness, self-efficacy, and academic anxiety, underscoring the necessity for diverse and targeted interventions to support student well-being during periods of crisis. Educational institutions should consider implementing stress management programs, enhancing academic and social support systems, and tailoring interventions to cater to different student demographics. These efforts can ensure that students remain engaged, connected, and supported throughout their educational experiences. By addressing these critical aspects, universities can mitigate the adverse effects of stress and anxiety, ultimately improving academic performance and fostering a stronger sense of community among students.

### 5.2 Limitations and future research

The present study is subject to a significant limitation due to its cross-sectional nature, which precludes the establishment of causal relationships between the variables in the model. Cross-sectional designs collect data at a singular point in time, which poses challenges in establishing the temporal order necessary for drawing causal conclusions (Wang and Cheng, 2020). For example, while structural equation modelling may suggest that stress related to COVID-19 adversely affects collegiate community belonging, it remains unclear whether the increase in stress leads to a decline in belonging or if a deficit in belonging triggers heightened stress levels. These relationships may be misinterpreted without longitudinal data, as causality cannot be definitively established (Maxwell et al., 2011). These variables may exert reciprocal influences. Factors such as loneliness and academic anxiety likely have mutual effects and interact with additional elements. While cross-sectional designs can provide insights into these complex relationships, they cannot conclusively determine whether loneliness exacerbates academic anxiety or whether increased anxiety results in feelings of loneliness. The interplay between these variables may create a feedback loop that is not observable without repeated measures over time. Furthermore, cross-sectional designs do not account for unobserved variables that may influence the relationships being studied (Setia, 2016). Additionally, the conclusions drawn from a cross-sectional study are often context-specific and may not generalise to different populations or settings (Wang and Cheng, 2020). For instance, the impact of COVID-19 stress on collegiate community belonging may vary significantly across diverse academic environments or among different demographic cohorts. Hence, interpreting these findings must acknowledge that the identified relationships may lack validity in longitudinal contexts or across varied settings. Consequently, it is imperative to exercise caution when inferring causal relationships among the variables due to the study’s cross-sectional design. Future investigations may yield valuable insights by adopting a longitudinal approach to more thoroughly explore the causal relationships affecting student well-being, particularly in relation to unique stressors such as the COVID-19 pandemic. Another notable limitation of this research is the reliance on convenience sampling, which may constrain the generalisability of the findings (Golzar et al., 2022). While efforts were made to create a diverse sample, the possibility of sampling bias cannot be entirely ruled out. Future research could address this limitation by utilising stratified sampling to ensure adequate representation of all subgroups within the population. Additionally, forthcoming studies might benefit from including other variables influencing students’ sense of belonging to their collegiate community and degree departments. Such variables could potentially mediate the relationships between stress resulting from the COVID-19 pandemic, academic anxiety, and feelings of loneliness. Furthermore, it is advisable to conduct data collection involving a more extensive and diverse student population. Lastly, future investigations should include students from various colleges and universities to comprehensively understand the relationships among these variables across different student demographics.

## Data Availability

The original contributions presented in the study are included in the article/supplementary material, further inquiries can be directed to the corresponding author.

## Footnotes

^1^The Russell Group consists of 24 preeminent UK universities established to safeguard the autonomy of higher education institutions and to uphold rigorous standards in both pedagogy and research endeavours (Russell Group, 2023).
