# Supplementary material for: Student well-being: the impact of belonging, COVID-19 pandemic related student stress, loneliness, and academic anxiety
Source: Front Psychol. 2025 Apr 28;16:1481328. doi: 10.3389/fpsyg.2025.1481328 (PMC12066278; doi:10.3389/fpsyg.2025.1481328)
Supplement: Supplementary file 1 [file Supplementary_file_1.docx]

Appendix 1.

| **Latent variable** | **Items** | | | **Manifest variable** | **Standardised Factor Loading** |
| --- | --- | --- | --- | --- | --- |
| *College Belonging* | |  |  |  |  |
|  | I have college-branded material that others can see (pens, notebooks, jumper, etc.). | | | CB1 | .658 |
|  | I tend to associate myself with my college. | | | CB2 | .860 |
|  | I would be proud to support my college in any way I can in the future. | | | CB3 | .827 |
|  | One of the things I like to tell people about is my college. | | | CB4 | .870 |
|  | I have found it easy to establish relationships at my college. | | | CB5 | .771 |
|  | I feel “at home” at my college. | | | CB6 | .874 |
|  | I attend college sporting events to support my college. | | | CB7 | .658 |
|  | My college provides opportunities to engage in meaningful activities. | | | CB8 | .753 |
|  | I feel similar to other people in my college. | | | CB9 | .776 |
|  | I feel connected to a staff member at my college. | | | CB10 | .700 |
|  | I feel a sense of pride when I meet someone from my college outside of university. | | | CB11 | .824 |
|  | I am proud to be a student at my college | | | CB12 | .880 |
|  | I believe that a staff member at my college cares about me. | | | CB13 | .697 |
|  | I take pride in wearing my college’s clothing. | | | CB14 | .858 |
|  | I feel that a staff member has appreciated me. | | | CB15 | .716 |
|  | I feel that a staff member has valued my contributions at my college. | | | CB16 | .783 |
|  | I feel like I belong to my college when I represent my college outside of university. | | | CB17 | .900 |
| *Department Belonging* | How well do people at your degree department understand you as a person? | | | DB1 | .818 |
|  | How connected do you feel to the academic staff at your degree department? | | | DB2 | .797 |
|  | How welcoming have you found your degree department to be? | | | DB3 | .839 |
|  | How much respect do other students at your degree department show towards you? | | | DB4 | .628 |
|  | How much respect do members of staff at your degree department show towards you? | | | DB5 | .767 |
|  | How much do you matter to others at your degree department? | | | DB6 | .786 |
|  | How happy are you with your choice to be a student at your degree department? | | | DB7 | .831 |
|  | How enriching is your experience at your degree department? | | | DB8 | .814 |
|  | How ‘at home’ do you feel at your degree department? | | | DB9 | .871 |
|  | Overall, how much do you feel like you belong to your degree department? | | | DB10 | .885 |
| *Loneliness* | I lack companionship. | | | LN1 | .695 |
|  | There is no one I can turn to. | | | LN2 | .734 |
|  | I feel left out. | | | LN4 | .756 |
|  | I feel isolated from others. | | | LN5 | .773 |
|  | I am unhappy being so withdrawn. | | | LN7 | .777 |
|  | People are around me but not with me. | | | LN8 | .804 |
|  |  | | |  |  |
| *Academic Anxiety* | I often worry that my best is not as good as expected in university. | | | AA1 | .766 |
|  | I tend to put off doing assignments because it stresses me. | | | AA2 | .707 |
|  | I often worry that I am not doing assignments properly. | | | AA3 | .803 |
|  | I am less confident about university than my peers. | | | AA4 | .811 |
|  | I have a sense of dread when I am in my degree settings. | | | AA5 | .806 |
|  | I tend to find my degree staff intimidating. | | | AA6 | .721 |
|  | I spend much of my time at university worrying about what is next. | | | AA7 | .760 |
|  | There is something about university that scares me. | | | AA8 | .804 |
|  | I’m concerned about what my peers think about my abilities. | | | AA9 | .742 |
|  | I often feel sick when I need to work on an important assignment. | | | AA10 | .783 |
|  | I have a hard time handling university responsibilities. | | | AA11 | .823 |
| *COVID-19 Student Stress* | How do you perceive the relationships with your colleagues at your degree department have been impacted by this period of COVID-19 pandemic? | | | CSSQ1 | .907 |
|  | How do you perceive the relationships with your college peers have been impacted by this period of COVID-19 pandemic? | | | CSSQ2 | .886 |
|  | How do you perceive the relationships with the academic staff at your degree department have been impacted by this period of COVID-19 pandemic? | | | CSSQ3 | .885 |
|  | How do you perceive the relationships with your college staff have been impacted by this period of COVID-19 pandemic? | | | CSSQ4 | .880 |
|  | How do you perceive your academic studying experience has been impacted by this period of COVID-19 pandemic? | | | CSSQ5 | .845 |
| *Coping Self Efficacy* | Break an upsetting problem down into smaller parts. | | | CSE1 | .855 |
|  | Sort out what can be changed, and what cannot be changed. | | | CSE2 | .805 |
|  | Make a plan of action and follow it when confronted with a problem. | | | CSE3 | .806 |
|  | Make unpleasant thoughts go away. | | | CSE4 | .912 |
|  | Take your mind off unpleasant thoughts. | | | CSE5 | .918 |
|  | Stop yourself from being upset by unpleasant thoughts. | | | CSE6 | .921 |
|  | Keep from feeling sad. | | | CSE7 | .813 |
|  | Get emotional support from friends and family. | | | CSE8 | .858 |
|  | Make new friends. | | | CSE9 | .841 |
